# *In vivo* spatiotemporal mapping of proliferation activity in gliomas via water-exchange dynamic contrast-enhanced MRI

**DOI:** 10.7150/thno.108479

**Published:** 2025-03-24

**Authors:** Ruiliang Bai, Yinhang Jia, Bao Wang, Zejun Wang, Guangxu Han, Lijun Liang, Lin Chen, Yang Ming, Guidong Zhu, Yi-Cheng Hsu, Peng Zhao, Yi Zhang, Zhiqiang Liu, Chong Liu, Zhaoqing Li, Yingchao Liu

**Affiliations:** 1 Interdisciplinary Institute of Neuroscience and Technology & Liangzhu Laboratory, Zhejiang University School of Medicine, Hangzhou, China.; 2 Key Laboratory of Novel Targets and Drug Study for Neural Repair of Zhejiang Province, School of Medicine, Hangzhou City University, Hangzhou, China.; 3 MOE Frontier Science Center for Brain Science and Brain-machine Integration, State Key Laboratory of Brain-machine Intelligence, Zhejiang University, Hangzhou, China.; 4 Key Laboratory of Biomedical Engineering of Ministry of Education, College of Biomedical Engineering and Instrument Science, Zhejiang University, Hangzhou, China.; 5 Lingang Laboratory, Shanghai, China.; 6 Department of Radiology, Qilu Hospital of Shandong University, Jinan, China.; 7 Department of Neurosurgery, Shandong Provincial Hospital Affiliated to Shandong First Medical University, Jinan, China.; 8 Department of Neurosurgery, Affiliated Hospital of Southwest Medical University, Luzhou, China.; 9 Department of Neurosurgery, The Second Hospital of Shandong University, Jinan, China.; 10 MR Collaboration, Siemens Healthcare, Shanghai, China.; 11 Department of Information, Shandong Provincial Maternal and Child Health Care Hospital Affiliated with Qingdao University, Jinan, China.; 12 MOE Frontier Science Centre for Brain Science and Brain-machine Integration, School of Brain Science and Brain Medicine, Zhejiang University, Hangzhou, China.; 13 Department of Physical Medicine and Rehabilitation of the Affiliated Sir Run Run Shaw Hospital AND Interdisciplinary Institute of Neuroscience and Technology, Zhejiang University School of Medicine, Hangzhou, China.; 14 Shandong Institute of Brain Science and Brain-inspired Research, Shandong First Medical University, Jinan, China.

**Keywords:** glioma, proliferation activity, water-exchange MRI, spatiotemporal monitoring

## Abstract

Proliferation activity mapping is crucial for the guidance of first biopsy and treatment evaluation of gliomas due to the highly heterogenous nature of glioma tumor. Here we propose and demonstrate an ease-of-use way of *in vivo* spatiotemporal mapping of proliferation activity by simply tracking transmembrane water dynamics with magnetic resonance imaging (MRI). Specifically, we demonstrated that proliferation activity can accelerate the transmembrane water transport in glioma cells.

**Method:** The transmembrane water-efflux rate (*k*_io_) measured by water-exchange dynamic contrast-enhanced (DCE) MRI. Immunofluorescence, immunohistochemistry, and immunocytochemistry staining were used to validate results obtained from the *in vivo* imaging studies.

**Results:** In glioma cell cultures, *k*_io_ precisely followed the dynamic changes of proliferation activity in growth cycles and response to temozolomide (TMZ) treatment. In both animal glioma model and human glioma, *k*_io_ linearly and strongly correlated with the spatial heterogeneity of intra-tumoral proliferation activity. More importantly, proliferation activity predicted by the single MRI parameter *k*_io_ is much more accurate than those predicted by state-of-the-art methods using multimodal standard MRIs and advanced machine learning. Upregulated aquaporin 4 (AQP4) expression were observed in most proliferating glioma cells and the knockout of AQP4 could largely slow down proliferation activity, suggesting AQP4 is the potential molecule connecting MRI-*k*_io_ with proliferation activity.

**Conclusion**: This study provides an ease-of-use, accurate, and non-invasive imaging method for the spatiotemporal monitoring of proliferation activity in glioma.

## Introduction

The malignancy of gliomas is largely attributed to heterogeneous tumor proliferation 1. *In vivo* mapping of the intratumoral heterogeneity of proliferation activity in gliomas is crucial for ensuring that the initial biopsy can accurately grade the tumor for precise treatment 2. Spatiotemporal mapping of proliferation activity in gliomas also facilitates timely evaluation of the treatment response and supports glioma recurrence monitoring 3-5, since radiotherapy, chemotherapy (*e.g.*, temozolomide [TMZ]), and the newly developed tumor treatment field of gliomas all target proliferative tumor cells, while silent, stem-like tumor cells often show treatment resistance and are believed to be the main sources of glioma recurrence 6,7.

Ki67 is the most widely used marker of cell proliferation in clinics 8. However, the extent of Ki67 expression can only be determined after surgical resection or biopsy of the tumor, making it impossible to assess Ki67 expression *in vivo*. Furthermore, Ki67 expression in gliomas exhibits considerable spatial heterogeneity, the pathological specimen used for analysis represents only a portion of the tumor tissue, leaving the expression of Ki67 in other tumor subregions unknown. Magnetic resonance imaging (MRI), free from ionizing radiation, plays a crucial role in managing gliomas. However, the standard MRIs implemented for glioma management lack sensitivity and specificity in detecting proliferation activity. Therefore, a non-invasive and high spatial-resolution method for Ki67 mapping could greatly improve the accuracy of glioma diagnosis and treatment monitoring.

**Figure 1:**
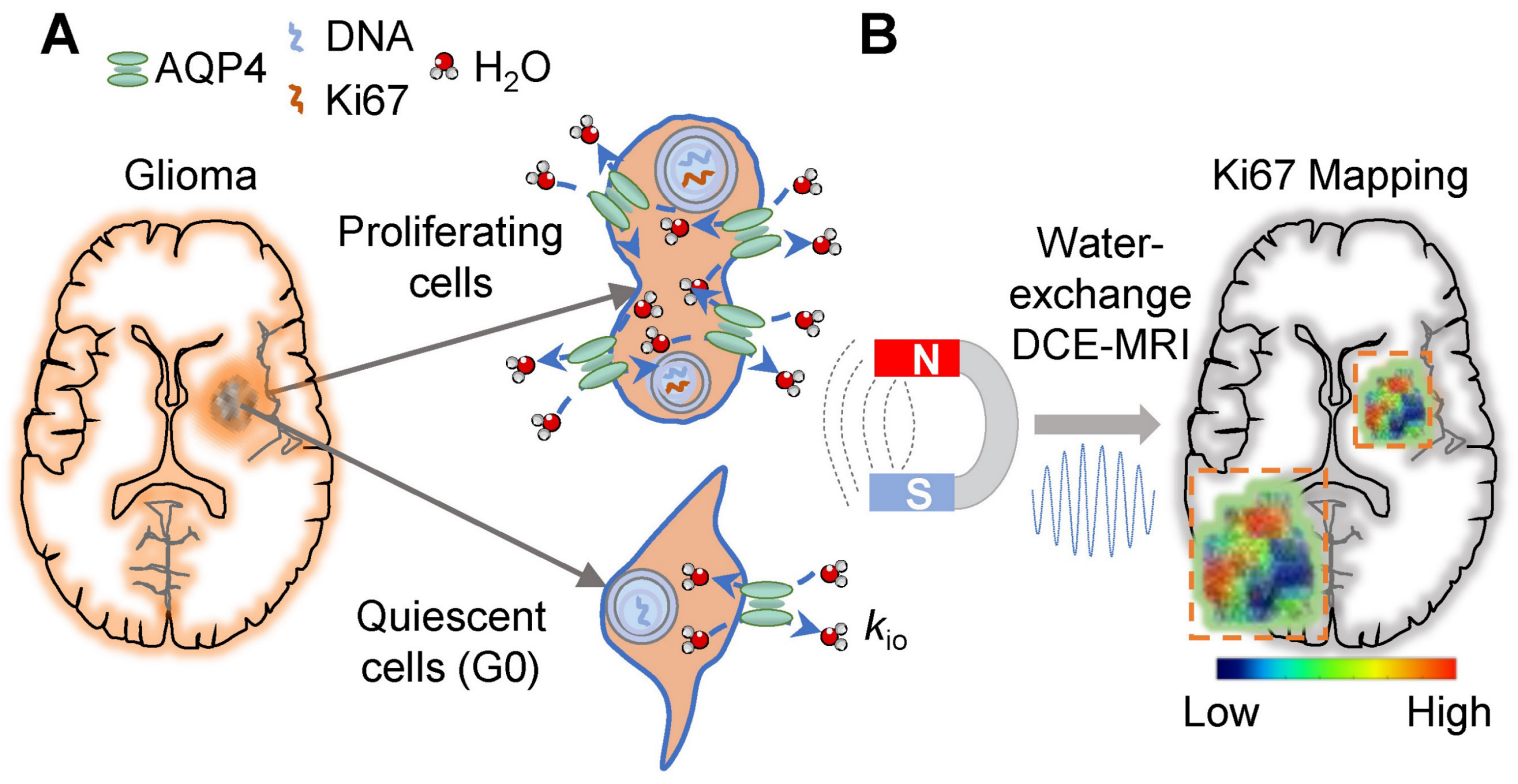
** Proliferation activity mapping of glioma via tracking water dynamics within MRI**. **A:** Proliferating activity can accelerate the transmembrane transport of molecules including water, and AQP4 upregulation during proliferation is the potential mechanism. **B:** The water-exchange DCE-MRI parameter, *k*_io_, is proposed as a linear surrogate of the proliferation activity, *i.e.*, Ki67 level.

Here we propose and show proliferation activity can accelerate the transmembrane water transport in glioma cells, which opens a new window for spatiotemporal mapping of proliferation activity in glioma by using water-exchange dynamic contrast-enhanced (DCE) MRI (**Fig 1**). Proliferation activity is a metabolically demanding process that requires the upregulation of molecule/ion transport across cell membranes, including water transport to maintain isotonicity 9,10. Water-exchange DCE-MRI, as with conventional DCE-MRI, which has been widely applied in pre-clinical and clinical studies, can be acquired via clinical widely used gadolinium-based contrast agents (CAs), *e.g.*, gadoteridol, and be combined with contrast-enhanced MRI for glioma diagnosis without added costs 11. Water-exchange DCE-MRI modifies the scan parameters of DCE-MRI to achieve higher sensitivity to transmembrane water exchange, and incorporates the shutter speed (SS) model in the analysis of DCE-MRI data, thus including transmembrane-water-exchange measurements into DCE-MRI 11-13.

Specifically, we show that the intracellular-to-extracellular transmembrane water-efflux rate (*k*_io_) obtained from water-exchange DCE-MRI is a unique and sensitive biomarker of proliferation activity in glioma (**Figure 1**). The accuracy of *k*_io_ as a surrogate of proliferation activity has been demonstrated by measurements of intertumoral heterogeneity of proliferation activity in both glioma animal models and human glioma with the help of stereotactic biopsy. Additionally, *k*_io_ was highly sensitive in tracking the dynamic changes in proliferation activity of glioma cell cultures, following a proliferation cycle and in response to TMZ. More importantly, the proliferation activity index, Ki67, predicted by the single MRI parameter *k*_io_, was much more accurate than those predicted by state-of-the-art methods using multimodal standard MRIs and advanced machine learning 14. Aquaporin 4 (AQP4) as the potential molecule connecting *k*_io_ to proliferation activity was also explored.

## Results

### *k*_io_ precisely captures the dynamic proliferation activity of glioma cells in a growth cycle and in response to TMZ treatment

**Figure 2:**
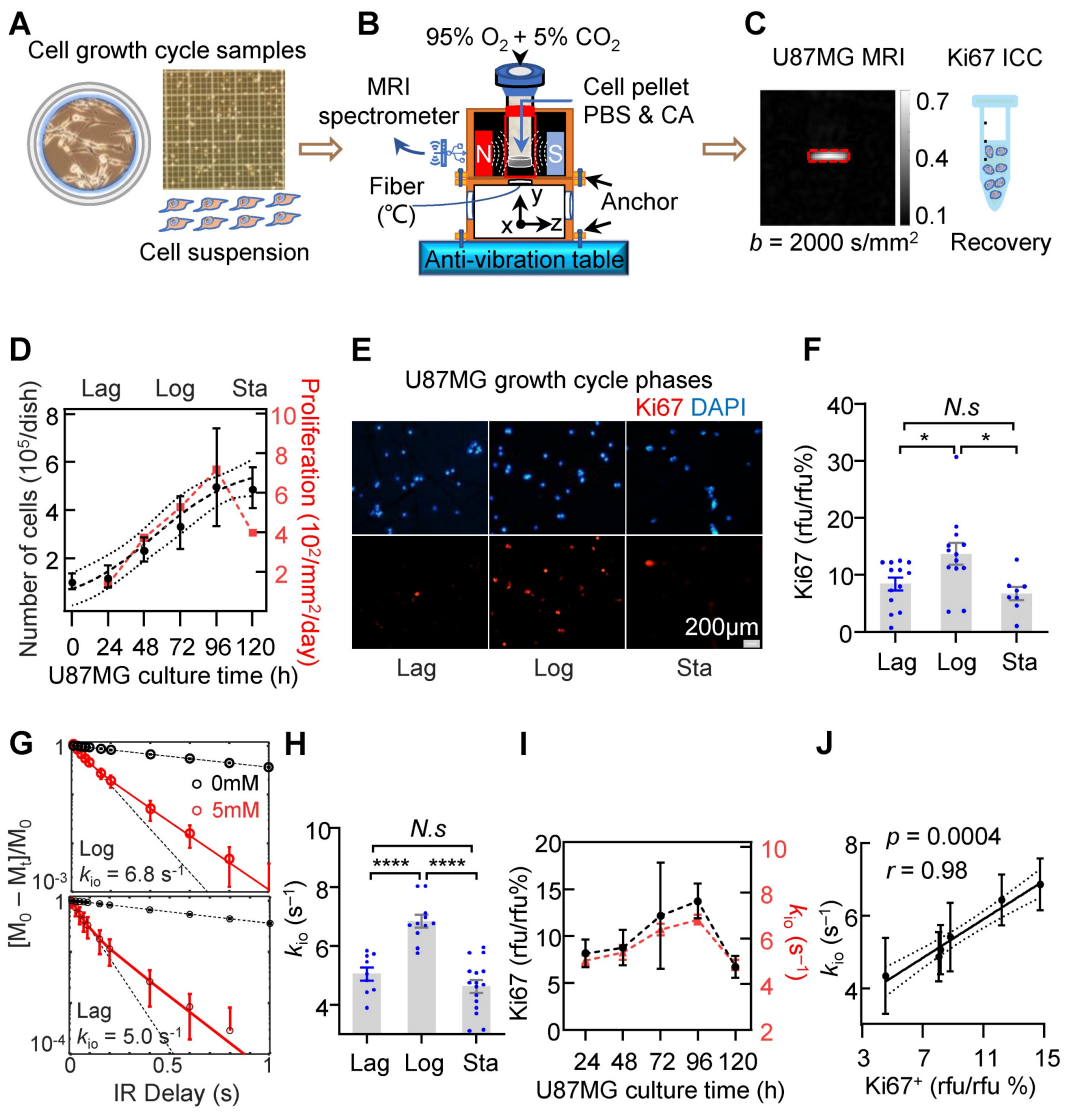
** MRI-*k*_io_ captures the dynamic proliferation activity of U87MG cells in a growth curve. A-C:** Experimental workflow.** A:** Preparation of U87MG samples with varying proliferation rates for MRI measurement, including cell culturing, the preparation of cell suspensions, and the counting of cell numbers at cell growth stages. **B:** Benchtop MRI system for cell culture measurement. The MRI system was mounted on an anti-vibration table, and a customized chamber was built for cell culture measurements, together with temperature monitoring via fiber-optic probe. Cells were collected and prepared as cell pellet for MRI measurement. The MRI spectrometer is utilized to receive signals from cells. **C:** A representative image of diffusion-weighted MRI to localize cell pellet (red rectangle), and the same samples used in MRI were collected for further Ki67 fluorescence (immunocytochemistry, ICC) analysis. **D:** U87MG cellular-growth curve fitted with the logistic equation: Y = 3.13/(4.56*exp(-0.04*Tc) + 0.61), goodness of fit *r* = 0.74, where Y mean Number of cells (10^5^/dish), Tc mean culture time (h). The data is shown as geometric mean (dots) with 95% confidence interval of mean. n = (10, 11, 8, 9, 8, 16). The right-hand Y-axis mean cell proliferation rate (red dots). Here the three proliferation phases were defined as Lag phase (24-48h), Log phase (72-96h), and Sta phase (from 120h). **E:** Ki67 expression in the samples after MRI during the Lag, Log and Sta phases for DAPI (blue, top) and Ki67 (red, bottom). **F:** Ki67 expressions in three phases, n = (13, 13, 8). one-way ANOVA tests *p < 0.05, *N.s*, non-significant. **G:** Demonstration of 2SX model fitting (continuous curve) on the normalized water-exchange DCE-MRI data at different CA concentrations and red dots for 5 mM. The black dashed curve is the result from a single-exponential fit, and its divergence from experimental data suggests that there is an intracellular water compartment with a longer *T*_1_ than the extracellular medium. **H:** *k*_io_ in three phases, n = (9, 11, 16), one-way ANOVA tests, ****p < 0.0001.* N.s*, non-significant. **I:** The dynamic changes in cellular Ki67 expression (black dots, n = 8, 5, 5, 13, 8) and *k*_io_ (red dots, n = 9, 8, 7, 11, 9) as functions of cell culture time. For **F, H, I**, the bar height and error bar width represent the mean and standard error of the mean (SEM), respectively. **J:** The correlation between *k*_io_ and Ki67 in the U87MG cell cycle. “rfu” denotes the quantitative fluorescence abundance results, *r* = 0.98, *p* = 0.0004, n = 51, the data were obtained from U87MG cell samples cultured for durations ranging 24-120h. The solid line indicates the linear regression, while the area between the two dashed curves represents the 95% confidence interval.

To assess the reliability of water-exchange DCE-MRI-derived *k*_io_ (MRI-*k*_io_) in monitoring the dynamic proliferation activity in glioma, water-exchange DCE-MRI measurements were performed on U87MG at different growth phases in which cells exhibit different proliferation status. In the depicted workflow (**Figure 2A-C**), we used the benchtop MRI system to measure cell pellets *in vitro* (**Figure S1A**) 11. Water-exchange DCE-MRI and cellular-growth curve measurements were taken on U87MG cells every 24 hours following sub-culturing, until the U87MG cell count in the dish stopped increasing. We observed a typical proliferation curve that displays a sigmoid shape (**Figure 2D,** goodness of fit: *r* = 0.74) and can be divided into the Lag phase, where cells adapt to their new environment after reseeding and the growth is slow, the Logarithmic (Log) phase, characterized by exponential growth, and the Stationary (Sta) phase where growth nearly ceases. The Ki67 expression levels peaked in the Log phase (72-96h, 13.7% ± 1.9%, n = 13) and is decreased by 39.1% in Lag phase (24-48h, 8.4% ± 1.1%, n = 13, *p* = 0.0355) and by 51.1% in Sta phase (from 120h, 6.7% ± 1.2%, n = 8, *p* = 0.0139) (**Figure 2E, F**).

To accurately estimate *k*_io_, we followed the experimental procedures in Jia *et al.* 11. Briefly, an inversion-recovery turbo spin echo (IR-TSE) sequence to quantitatively measure longitudinal relaxation time (*T*_1_) were acquired at two CA concentrations and a two-site (intracellular and extracellular) exchange (2SX) model was applied to estimate *k*_io_ (**Figure S1B**). Here, a clinical routinely used Gd-based extracellular CA (Prohance, Bracco Diagnostics) was used to label the extracellular water with shorter *T*_1_. The 2SX model well described the IR-TSE signal in different phases and the obtained *k*_io_ in the Log phase of samples are much larger than that in the Lag phase (**Figure 2G**). Statistically, *k*_io_ in the Log phase (6.8 ± 0.7 s⁻¹, n = 11) is significantly larger than those in the Lag phase (5.0 ± 0.7s⁻¹, n = 9, *p* < 0.0001) and in the Sta phase (4.6 ± 0.9 s⁻¹, n = 16, *p* < 0.0001) (**Figure 2H**).

Interestingly but unsurprisingly, MRI-*k*_io_ and Ki67 expression exhibited a similar trend across cell growth cycles (**Figure 2I**). Further analysis demonstrated a significant and strong correlation between Ki67 and MRI-*k*_io_ using the data obtained from each day (**Figure 2J**, correlation coefficient* r* = 0.98, *p* = 0.0004). These results demonstrated that MRI-*k*_io_ precisely captured the dynamic proliferation activity of glioma cell cultures in the cell growth cycles.

**Figure 3:**
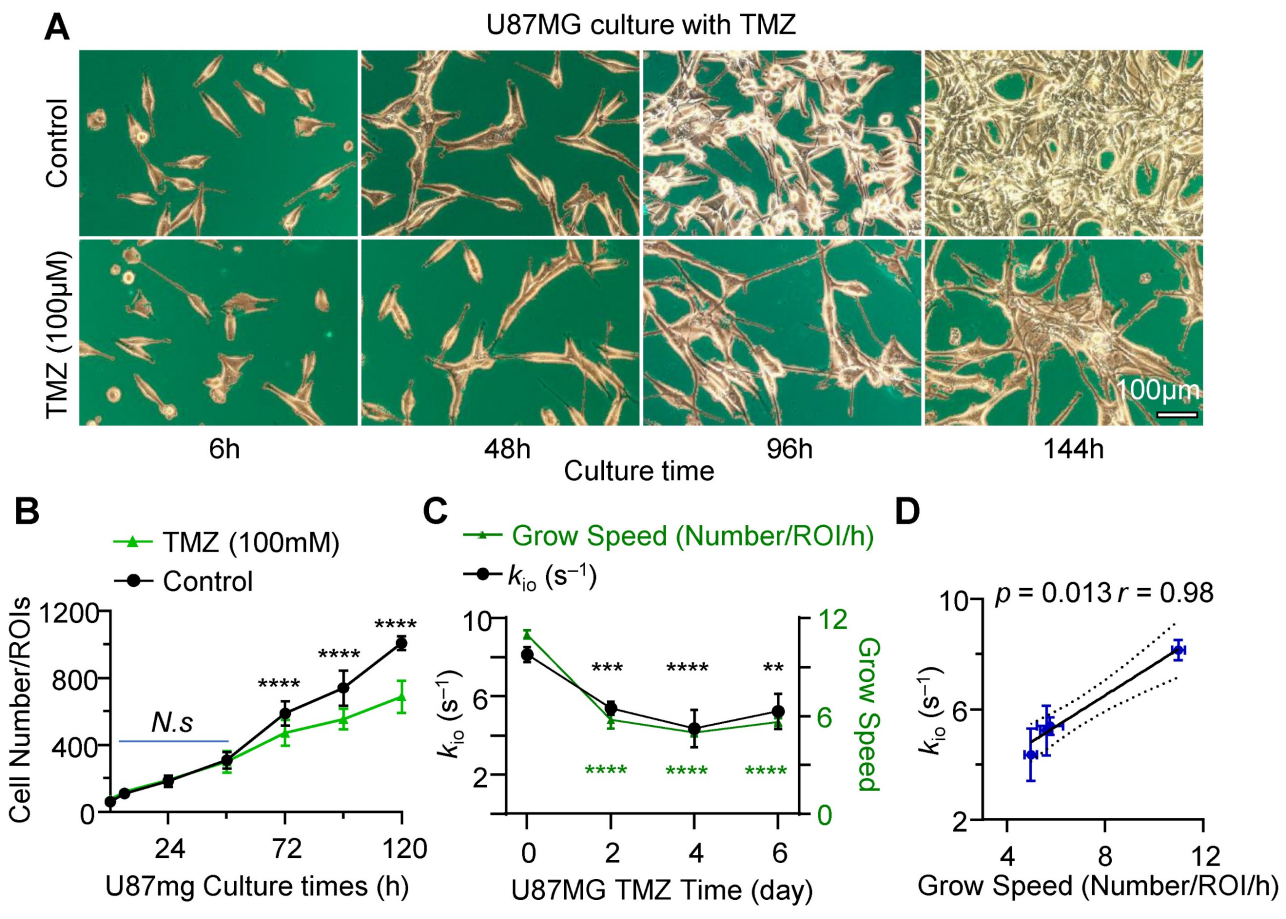
** MRI-*k*_io_ captures the dynamic proliferation activity of U87MG cells in response to TMZ treatment. A:** Morphology of U87MG cells during TMZ treatment, with cells in brown color and culture dish marked with blue color, Scale bar, 100 μm. **B:** Growth curve of the U87MG cells with TMZ treatment (n = 11) and the control group (n = 12) were represented by green dots and black dots. ****p < 0.0001. **C:** The dynamic changes in cellular growth speed (right Y-axis, green dots, n = 8, 4, 4, 4) and *k*_io_ (left Y-axis, block dots, n = 5, 8, 3, 3) as functions of TMZ culture time. Here, the X-axis starts at 0, representing the control group (untreated U87MG cells in Log growth phase), **p < 0.01, ***p < 0.001, ****p < 0.0001. For **B** and **C** two-way ANOVA Multiple comparisons results between Control and TMZ group. **D:** Correlation analysis between *k*_io_ and cell growth speed, utilizing data from U87MG cells at various TMZ treatment durations (TMZ = 0, 2, 4, 6 days, n = 5, 8, 3, 3), *r* = 0.98, *p* = 0.013. The solid line indicates the linear regression, while the area between the two dashed curves represents the 95% confidence interval. For **B, C** and **D**, data are presented as mean ± SEM.

To further demonstrate the capability of water-exchange DCE-MRI in detecting the dynamic change of proliferation activity, TMZ treatment was performed by incubating the U87MG cell line with 100 μM TMZ dissolved in 0.1% dimethyl sulfoxide (DMSO). Time-lapse microscopy showed that TMZ successfully suppressed the growth of glioma cells in comparison to the control group (only 0.1% DMSO) (**Figure 3A**). The inhibitory effect of TMZ on the proliferation of U87MG glioma cells was quantified by measuring the number of cells per unit area. It showed a substantial decrease in the number of cells (**Figure 3B**). Notably, commencing from the 72h post-treatment, TMZ markedly reduced the number of cells within region of interest (ROI). The number of cells within the ROI under TMZ treatment (n = 11), with values of 472.2 ± 22.7 for 72h, 553.7 ± 19.3 for 96h, and 687.8 ± 29.1 for 120h, exhibited a significant decrease of 24.2% (*p* = 0.0012), 33.3% (*p* < 0.0001), and 46.5% (*p* < 0.0001) compared to the control group (586.7 ± 20.6, 738.4 ± 30.3, 1007.3 ± 11.8, n = 12), respectively. In **Figure 3C**, the growth speed of 0-day TMZ treatment (with samples derived from Log cells), was significantly higher than that observed on 2^nd^ day, 4^th^ day, and 6^th^ day of TMZ treatment. The growth speed on 0-day (11.0% ± 0.3%, n = 8) increased significantly by 47.3% (*p* < 0.0001), 54.5% (*p* < 0.0001), and 49.0% (*p* < 0.0001) compared to the growth speed on 2^nd^ day (5.8% ± 0.5%, n = 4), 4^th^ day (5.0% ± 0.2%, n = 4), and 6^th^ day (5.6% ± 0.2%, n = 4) of TMZ treatment (**Figure 3C**, black), respectively.

Further water-exchange DCE-MRI measurements revealed that *k*_io_ on the TMZ 0-day (8.1 ± 0.4 s⁻¹, n = 5) was 24.2% (*p* = 0.0002) larger than that on the 2^nd^ day (5.4 ± 0.3s⁻¹, n = 8) of TMZ, was also 33.3% larger (*p* = 0.0044) than that on the 4^th^ day (4.4 ± 1.0 s⁻¹, n = 3) and 46.5% larger (*p* = 0.0125) than that on the 6^th^ day (5.2 ± 0.9s⁻¹, n = 3) (**Figure 3C**, green). Additionally, a significant linear correlation was observed between *k*_io_ and growth speed during TMZ treatment in both U87mg cells (**Figure 3D**, *r* = 0.98, *p* = 0.013) and rat glioma C6 cells (**Figure S2**, *r* = 0.99, *p* = 0.0003), suggesting MRI-*k*_io_ as a biomarker for TMZ treatment efficacy.

### MRI*-k*_io_ precisely detects intratumoral and intertumoral heterogeneity of proliferation activity in rat glioma models

**Figure 4:**
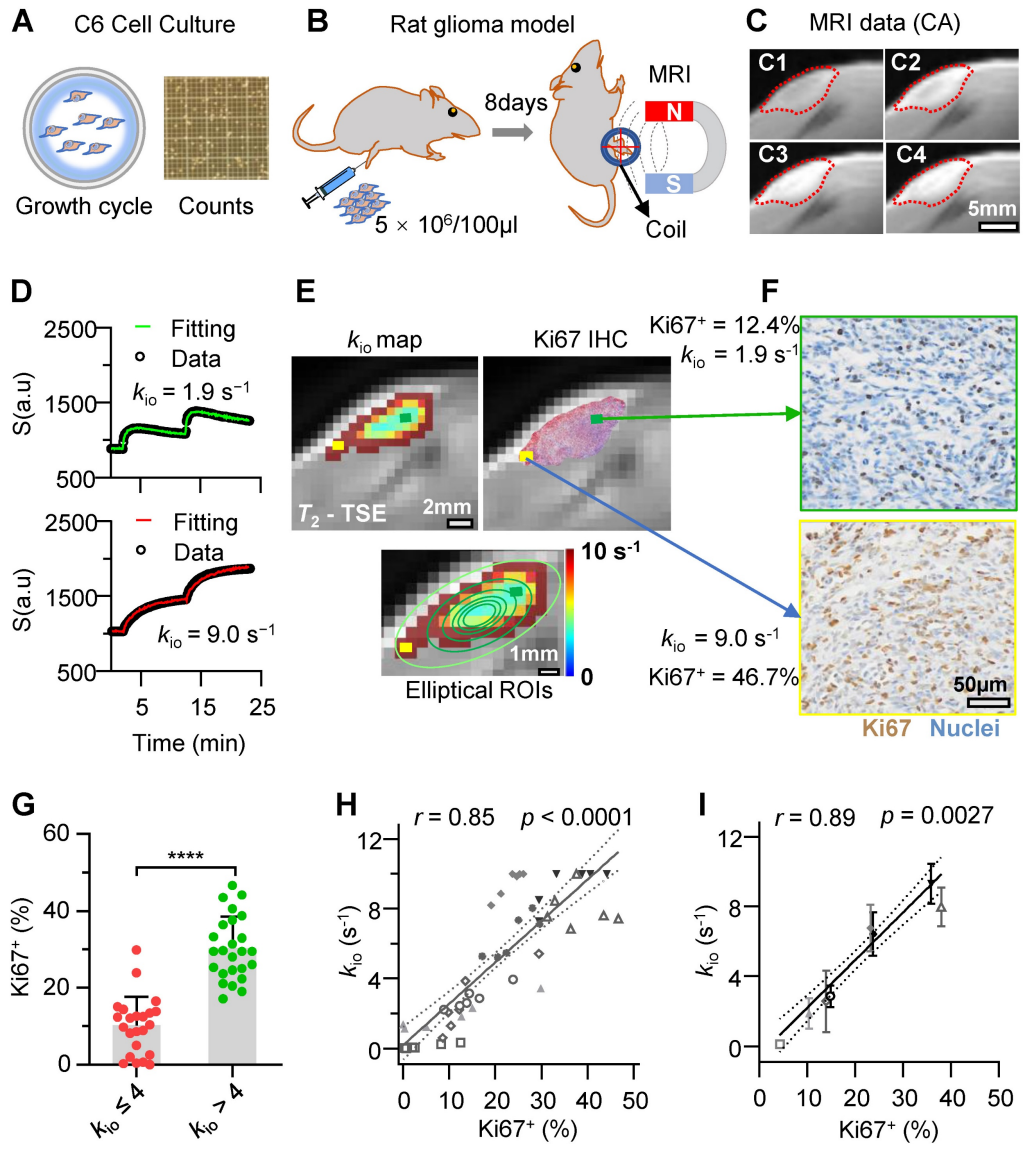
** Water-exchange DCE-MRI precisely detected the intratumoral and intertumoral Ki67 expression heterogeneity in rat glioma model. A-C:** Schematic of the experimental design; **A,** Rat glioma cells (C6) culture for *in vivo* models (**B**). **C,** water-exchange DCE-MRI raw images of a rat glioma model at Pre-CA (**C1**), post-CA 2 mins (**C2**), post-CA 10 minutes(**C3**), and the last frame (post-CA 24 minutes, **C4**) of the sequence, with the ROI of the tumor indicated in red. **D:** Representative results of the water-exchange DCE-MRI data fitted with the 3S2X model for tumor pixels (top, low *k*_io_ = 1.9 s^-1^; bottom, high* k*_io_ = 9.0 s^-1^). The raw data and fitting results are shown as dots and continuous curves, respectively.** E:** Top, the *k*_io_ maps (left) and the immunohistochemistry (IHC) results (right) were overlaid on the same *T*_2_-weighted images (rat from **C**). Ki67^+^ and cell nuclei are in red and green, respectively. Bottom, six contour lines were used to divide the tumor slice into six concentric donut-shaped ROIs per rat. **F:** Enlarged *k*_io_ results from different tumor pixel (yellow and green), whose positions are illustrated in **E**. **G:** Comparison of Ki67 results between low *k*_io_ (≤ 4s^-1^) elliptical ROIs and high *k*_io_ (> 4s^-1^) elliptical ROIs. n = (23, 25), unpair *t*-test, ****p < 0.0001. Data are presented as mean ± SEM. **H:** Linear correlation between the ROI-averaged *k*_io_ and Ki67^+^ cell fractions of 48 total ROIs (six ROIs per rat, n = 48; data from the same rat are displayed with the same symbols), *r* = 0.85, *p* < 0.0001. **I**: A similar linear correlation was observed between the whole-tumor-averaged *k*_io_ and the Ki67^+^ fractions (the same symbol was used for each rat as in **H**), *r* = 0.89, *p* = 0.0027, n = 8 (six ROIs per rat). For **H**, **I**, the solid line indicates the linear regression, while the area between the two dashed curves represents the 95% confidence interval.

To explore the capability of water-exchange DCE-MRI in detecting the spatial distribution of proliferation inside and across glioma tumors, water-exchange DCE-MRI was performed in a Sprague-Dawley rat model of subcutaneous C6 cell glioma (**Figure 4A-C**) with the GRE (Gradient-Echo) sequence and a clinically used Gd-based CA (gadopentetate dimeglumine, Condun, Guangzhou, China). Following the injection of the CA, the tumor region was largely enhanced (**Figure 4C** and **Supplementary Video**). For the majority of pixels, the water-exchange DCE-MRI data showed good quality and the three-site two-exchange (3S2X) model specifically developed for glioma tumor DCE-MRI analysis *in vivo* (see **Methods**) showed excellent fitting to the time-course data (**Figure 4D**). The additional site comparing to the 2SX model used in cell culture measurements is the vascular component *in vivo.* The *k*_io_ map (**Figure 4E**) and Ki67 expression of the corresponding slices showed similar spatial distributions on visual inspection, *e.g*., in **Figure 4E, F** higher* k*_io_ together with greater Ki67 expression in the tumor margin (yellow rectangle) was observed and vice versa (green rectangle).

To quantitatively evaluate the accuracy of Ki67 detected by water-exchange DCE-MRI *in vivo*, we used a series of concentric donut-shaped ROIs to divide the whole tumor slice into six zones (**Figure 4E,** bottom). Then, the *k*_io_ and the Ki67 positive fraction values (Ki67^+^) in histology were calculated for each corresponding ROI (calculation process shown in the **Methods**). Further statistical analysis revealed that elliptical ROI tissues with elevated *k*_io_ values (> 4 s⁻¹) had significantly higher Ki67 expression than those in ROIs with *k*_io_ ≤ 4 s⁻¹ (**Figure 4G**, 30.2% ± 8.2% vs 10.1% ± 7.5%, *p* < 0.0001, n = (23, 25)), suggesting a reliable association between *k*_io_ and Ki67 expression throughout glioma progression. Further Pearson correlation analysis revealed that the *k*_io_ had a significant and linear correlation with Ki67^+^ expression in rat gliomas, *k*_io_ = 24 s^-1^ × Ki67^+^ % + 0.20s^-1^ (**Figure 4H**, *r* = 0.85, *p* < 0.0001). A similar linear relationship existed between Ki67 expression and *k*_io_ (**Figure 4I**,* r* = 0.89, *p* = 0.0027) when the analysis was performed at the intertumoral level using the whole-tumor-averaged Ki67 expression and *k*_io_ in each rat.

### Human glioma biopsies with faster water-efflux rate exhibit more aggressive proliferation

**Figure 5:**
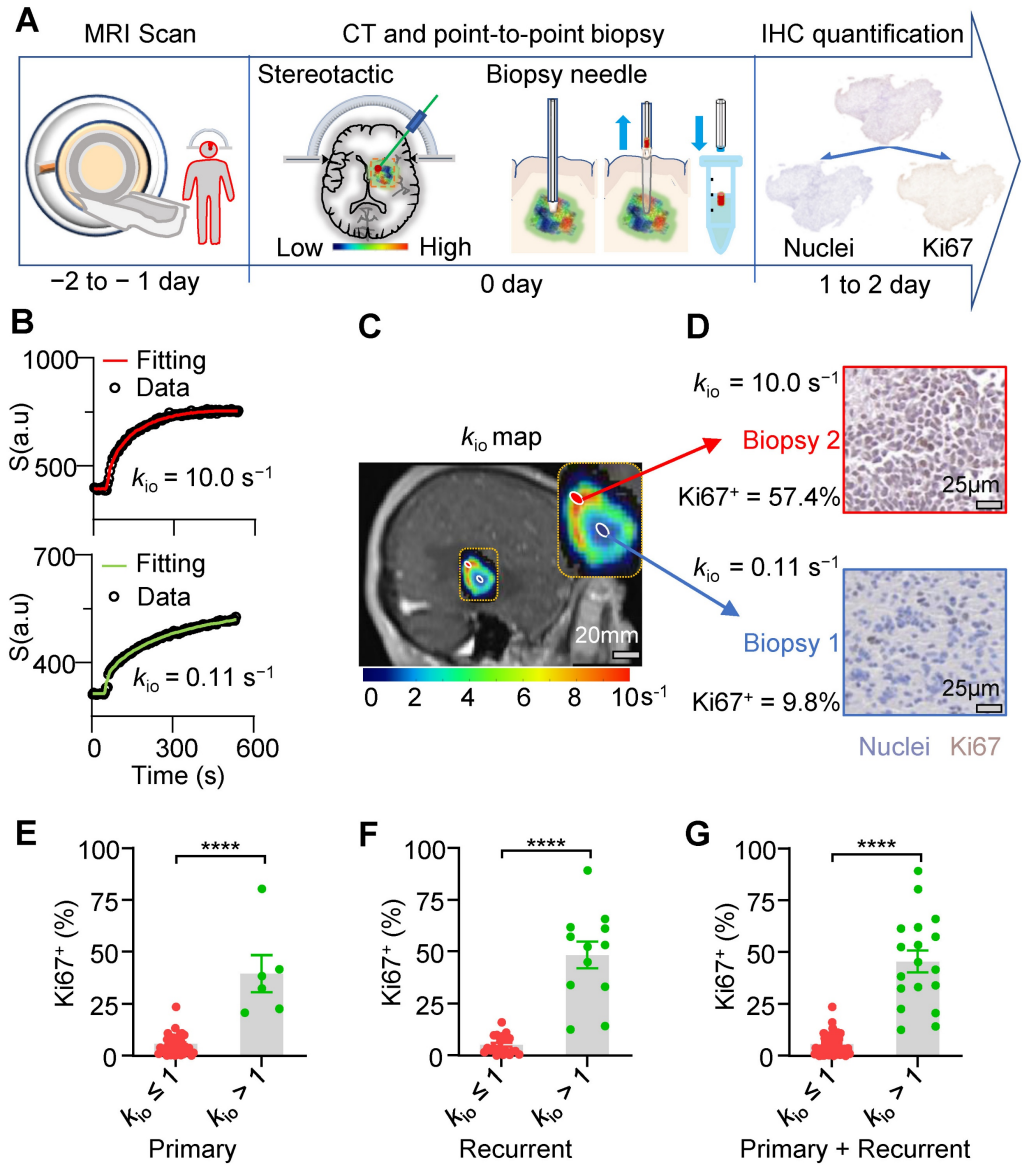
***k*_io_-guided stereotactic biopsy enables “point-to-point” comparison of MRI and histology. A:** Experimental workflow (from left to right): water-exchange DCE-MRI, stereotactic biopsy procedure for human gliomas, radiographic-histopathological point-to-point paired data and downstream molecular analysis.** B:** Representative results of the water-exchange DCE-MRI data fitted with the 3S2X model (see **methods**) for tumor biopsies (top, high *k*_io_ = 10.0 s^-1^; bottom, low *k*_io_ = 0.11 s^-1^). The raw data and fitting results are shown as dots and continuous curves, respectively.** C:** Illustration of a *k*_io_ map overlaid on the 70^th^ DCE-MRI frame, guiding two biopsy points acquired using the same needle and high (**D**, top) and low (**D**, bottom) *k*_io_ biopsies. **D:** On IHC, brown indicates Ki67^+^ cells; blue indicates nuclei; larger *k*_io_ values showed greater Ki67 expression and vice versa. **E-G:** Comparison of biopsy Ki67 results between high *k*_io_ (> 1 s^-1^) and low *k*_io_ (≤ 1 s^-1^) points. Data are presented as mean ± SEM. **E,** Primary patients, n = (40,6); **F,** recurrent patients, n = (21,12). **G**, all patients (primary and recurrent), n = (61,18). nonparametric tests (Mann-Whitney U test), ****, *p* < 0.0001.

Here, we further evaluated the ability of our exclusive water-exchange DCE-MRI to detect intratumoral heterogeneity of proliferation activity in human gliomas using point-to-point correlation analysis between the MRI-*k*_io_ and histological Ki67 expression. **Figure 5A** shows the workflow. Between May 2019 and June 2023, patients with suspected primary or recurrent brain gliomas undergoing biopsies were enrolled. Written informed consent was obtained from each participant. Before biopsy, water-exchange DCE-MRI was performed following the protocol of Bai at al. and Jia *et al.* 11,12. Briefly, the flip angle of DCE-MRI was optimized to improve the sensitivity to transmembrane water exchange and the widely used CA, 0.1 mmol pre-kg body weight, was used and injected at 8th frame of DCE-MRI. Intracellular water-efflux rate *k*_io_ maps were generated by fitting the water-exchange DCE-MRI data with the 3S2X model 12. This study was approved by our Institutional Review Board (LCYJ: NO. 2019-103). Overall, 79 biopsy samples from 34 participants (mean age, 52 years; age range, 12-76 years; 15 men) were included in this study (**Table S1 and Figure S3**). Tumor grading according to the 2021 WHO guidelines revealed that 7 participants had grade II glioma, 8 participants had grade III glioma, and 19 participants had grade IV glioma. **Table S2** and **Figure S3** provide further information about the patients and biopsy samples included in this study, in accordance with the STARD guidelines.

Most MRI pixels showed excellent fit (**Figure 5B**). We then merged the *k*_io_ maps into structural images to guide stereotactic biopsy with varying *k*_io_ values for each patient (**Figure 5C**). Values from different spatial biopsy points and paired IHC were then calculated. ROIs with higher *k*_io_ consistently exhibited higher Ki67 expression in their corresponding biopsies. For example, biopsy samples displaying a *k*_io_ of 10 s^-1^ exhibited largely higher Ki67^+^ (57.4% vs 9.8%) compared with samples displaying a *k*_io_ of 0.11 s^-1^ in the same tumor (**Figure 5D**). Further statistical analysis revealed that biopsy tissues with elevated *k*_io_ values (> 1 s⁻¹) had significantly increased Ki67^+^ in both primary (**Figure 5E**, 39.5% ± 8.9% vs 5.8% ± 0.76%, *p* < 0.0001, n = (40,6)) and recurrent (**Figure 5F**, 48.5% ± 6.4% vs 5.3% ± 0.97%, *p* < 0.0001, n = (21,12)) gliomas. This trend was consistent across all patient biopsies (**Figure 5G**, 45.5% ± 5.1% vs 5.6% ± 0.60%, *p* < 0.0001, n = 79).

### A strong and linear correlation was evident between MRI-*k*_io_ and histology-Ki67^+^ in both primary and recurrent human glioma

**Figure 6:**
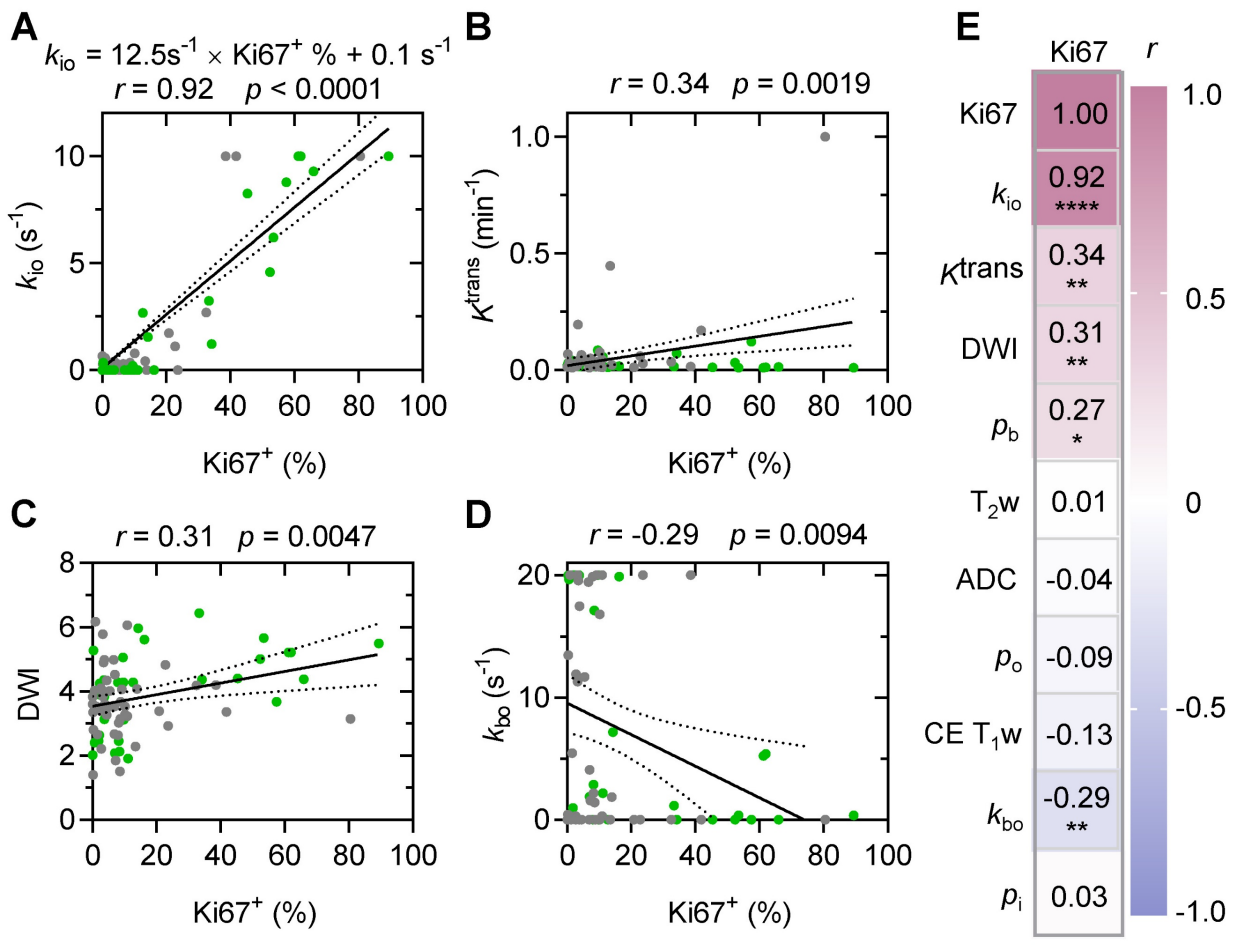
** MRI-*k*_io_ strongly and linearly correlates with biopsy Ki67^+^ in human gliomas. A:** MRI-*k*_io_ values and Ki67^+^ were linearly correlated in point-to-point biopsy samples (n = 79). The gray and green solid circles are biopsy data from the primary (n = 46) and recurrent gliomas (n = 33), respectively. **B-D:** Linear correlations of Ki67^+^ expression with *K*^trans^ (**B**), DWI (**C**), and *k*_bo_ (**D**).** E:** Visualized Pearson correlations between Ki67 and subsets of clinician-selected variables, * *p* < 0.05, ** *p* < 0.01, **** *p* < 0.0001. The solid line indicates the linear regression, while the area between the two dashed curves represents the 95% confidence interval. Gray and green solid circles represent biopsy data from primary and recurrent gliomas, respectively. Here, the water mole fractions for cells, blood and extracellular extravascular are defined as *p*_i_, *p*_b_, *p*_o_, respectively. The steady-state water molecule extravasation was *k*_bo_. *K*^trans^ denotes the contrast agent extravasation rate constant. CE T1w, contrast-enhanced T1-weighted images. T2w, T2-weighted images, ADC: apparent diffusion coefficient.

A strong and significant linear correlation (**Figure 6A,*** r* = 0.92, *p* < 0.0001) between MRI-*k*_io_ and histology Ki67^+^ was observed in both the primary human glioma (n = 46) and the recurrent human glioma (n = 33). Among all other MRI metrics, only contrast agent extravasation rate constant (*K*^trans^) from DCE-MRI (**Figure 6B**, *r* = 0.34; *p* = 0.002), diffusion weighted imaging DWI intensity (**Figure 6C**, *r* = 0.31; *p* = 0.005), vascular water efflux rate constant *k*_bo_ from DCE-MRI (**Figure 6D**, *r* = -0.29; *p* = 0.009), and vascular water mole fraction *p*_b_ from DCE-MRI (**Figure 6E,** *r* = 0.27; *p* = 0.015) showed significant but weak correlations with Ki67^+^.

### Comparison with standard MRI modalities in predicting stereotactic biopsy Ki67^+^

**Figure 7:**
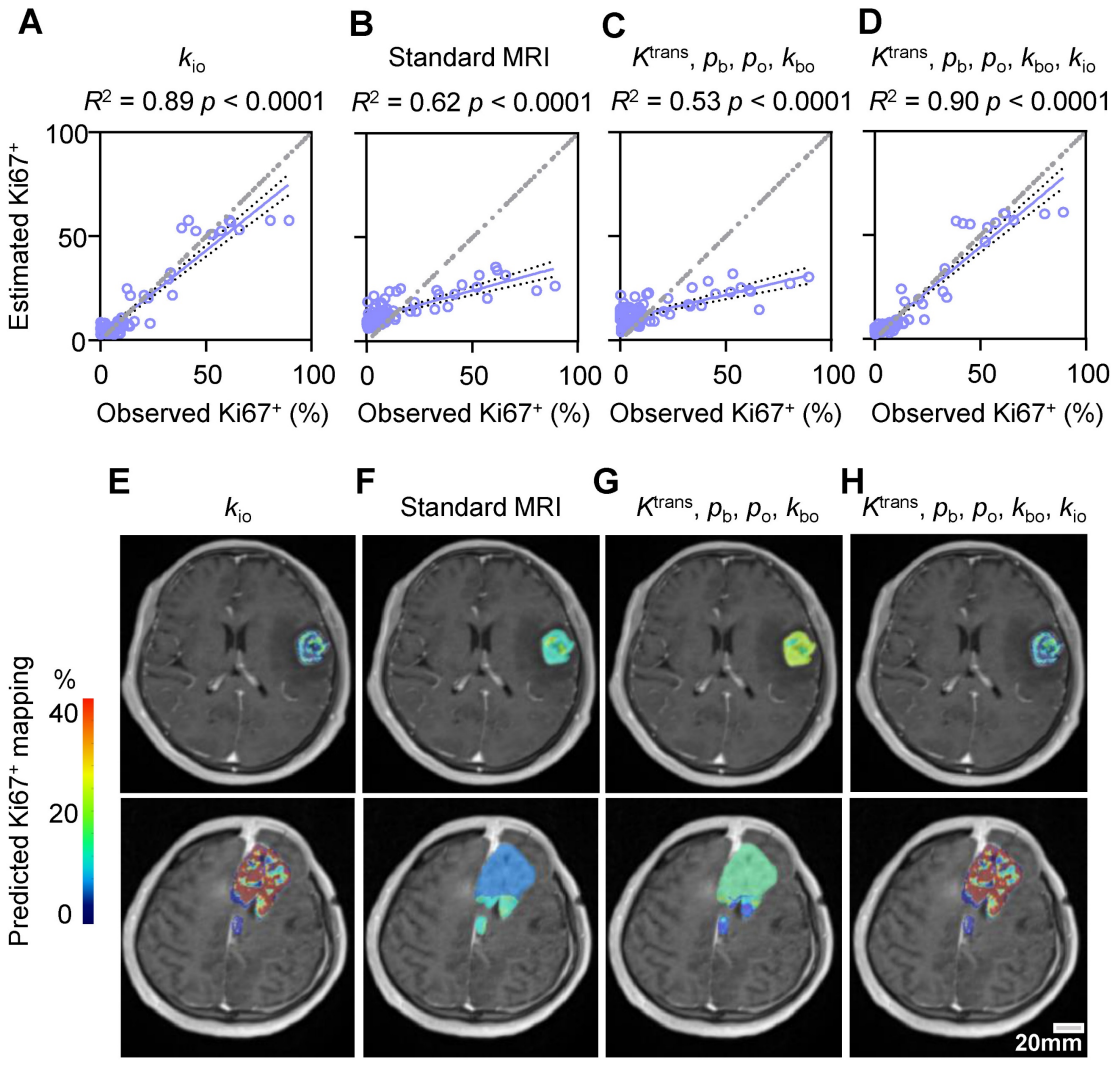
** Comparison with standard MRI modalities in predicting stereotactic biopsy Ki67^+^.** (**A-D**) Prediction results with a random forest model evaluated by five-fold cross validation of 79 stereotactic biopsy points using the MRI metric(s) of: single parameter *k*_io_ (**A**), standard MRI (T2w, DWI, ADC, and CE T1w) (**B**), four DCE-MRI-derived pharmacokinetic parameters (*K*^trans^, *k*_bo_, *p*_b_, and *p*_o_) (**C**), and five DCE-MRI-derived pharmacokinetic parameters (*K*^trans^, *k*_bo_, *p*_b_, *p*_o_, and *k*_io_) (**D**). The solid line indicates the linear regression, while the area between the two dashed curves represents the 95% confidence interval. (**E-H**) Two examples of the predicted Ki67 expression map in patients with WHO III (**up**) and WHO IV (**down**) glioma using the respective prediction models displayed in **A-D**.

We further compared the performances of standard MRI with multiple modalities and water-exchange DCE-MRI in predicting Ki67^+^ using the stereotactic biopsy (n = 79) data. Across all tested machine learning algorithms, *k*_io_ consistently performed the best, explaining 80% to 89% of the variance (**Table S3**). Among all machine learning models, the random forest model performed best in all datasets (**Table S3**). Utilizing this random forest model 2, the single parameter *k*_io_ could effectively predict Ki67^+^ with an *R*² of 0.89 and a root mean square (RMS) error of 6.9% (**Figure 7A, *p* < 0.0001**). In contrast, standard MRI with multiple modalities (T2w, DWI, ADC, and CE T1w) could only predict histology-Ki67^+^ with an *R*² of 0.62 and an RMS error of 15.1% (**Figure 7B, *p* < 0.0001**). The other water-exchange DCE-MRI parameters (*K*^trans^, *k*_bo_, *p*_b_, and *p*_o_) also could only predict histology-Ki67^+^ with an *R*² of 0.53 and an RMS error of 15.9% (**Figure 7C, *p* < 0.0001**). The combination of these other water-exchange DCE-MRI parameters with *k*_io_ only slightly improved the performance in predicting Ki67^+^ compared with the single parameter *k*_io_ (*R*^2^ from 0.89 to 0.90, RMS error from 6.9% to 6.7%, **Table S3**), highlighting the dominant role of *k*_io_ in predicting Ki67^+^ (**Figure 7A-D**).

### Predicted Ki67^+^ level maps in glioma tumors

Representative maps of the predicted Ki67^+^ using the random forest model with different MRI modalities were shown in **Figure 7E-H**. For subjective and visual assessment, *k*_io_-predicted Ki67^+^ maps exhibited spatial heterogeneity, whereas those predicted by standard MRIs and other water-exchange DCE-MRI-derived pharmacokinetic metrics showed a more homogeneous distribution within the tumor. Further quantitative analysis of high-grade glioma heterogeneity using the entropy metric revealed considerably higher intratumoral heterogeneity for Ki67^+^ maps predicted by *k*_io_ (1.11 ± 0.31) than for those predicted by standard MRIs (0.05 ± 0.01, *p* = 0.009, n = 27) and other water-exchange DCE-MRI-derived pharmacokinetic metrics (0.05 ± 0.01, *p* = 0.009, n = 27). Additionally, the *k*_io_-predicted Ki67^+^ maps could also capture inter-tumoral proliferation heterogeneity. For instance, patients with WHO IV tumors showed substantially higher Ki67 expression than patients with WHO III tumors (**Figure 7E, H**). This difference became much smaller in Ki67^+^ maps predicted by standard MRIs (**Figure 7F**) and other water-exchange DCE-MRI-derived pharmacokinetic metrics (**Figure 7G**). No obvious differences were observed between the Ki67^+^ maps predicted by *k*_io_ alone (**Figure 7E**) and those predicted by the combination of *k*_io_ and other water-exchange DCE-MRI-derived pharmacokinetic metrics (**Figure 7H**).

### AQP4 knockout (KO) largely slowed down the fraction of Ki67^+^ cells both *in vitro* and* in vivo*

**Figure 8:**
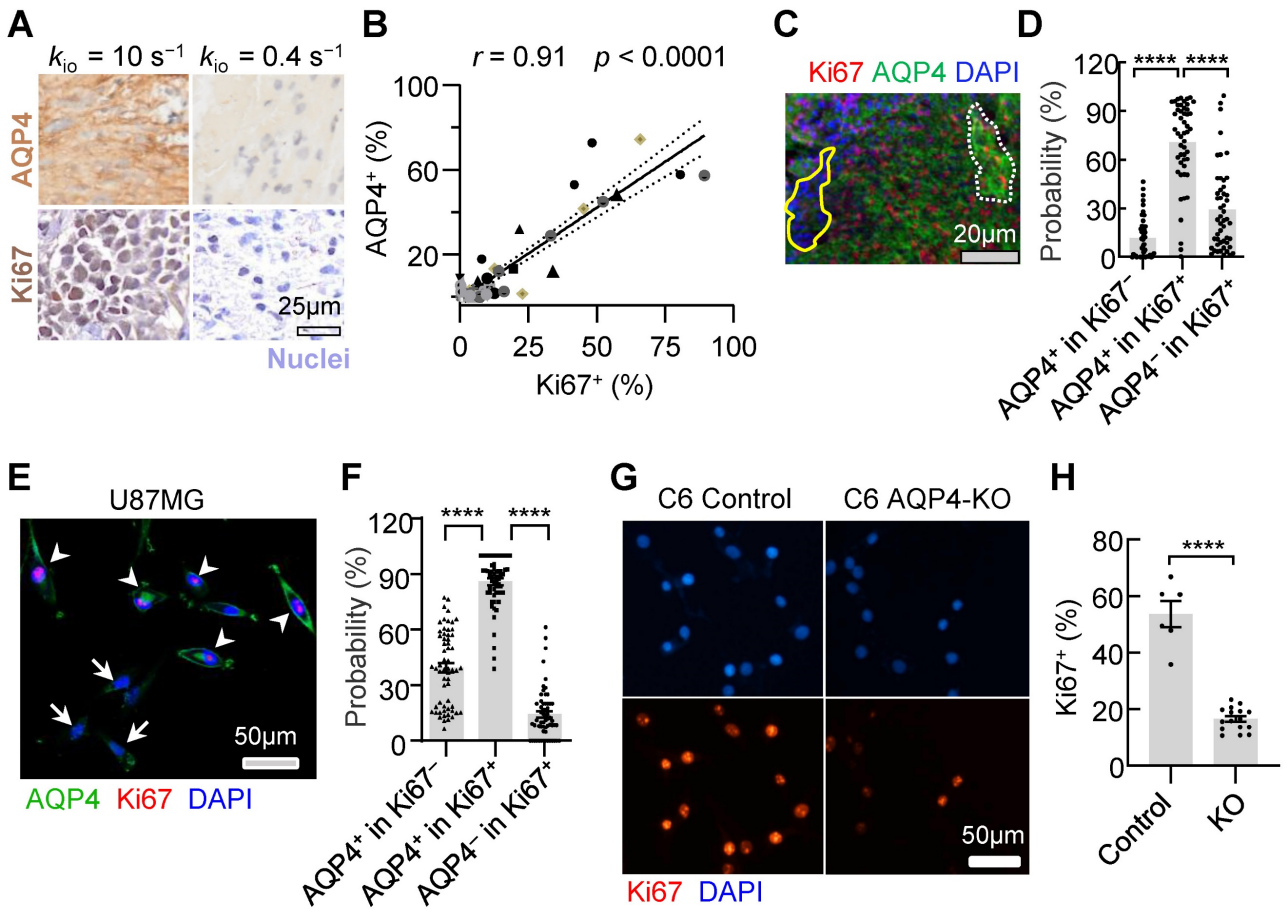
** Synchronous expression of Ki67 and AQP4 in glioma. A:** Typical examples of AQP4 (up) and Ki67 (down) IHCs of the stereotactic biopsy tissues with high (left) and low (right) *k*_io_, respectively.** B:** A linear correlation is observed between Ki67^+^ and AQP4^+^ cells in 55 stereotactic biopsy points. The solid line reflects linear regression analysis and the two dashed curves denote 95% confidence intervals.* r* = 0.91, *p* < 0.0001. **C:** Multiplex IHC results (AQP4: green; Ki67: red; DAPI: blue). Dotted white lines indicate areas where both typical AQP4 and Ki67 were expressed. The region enclosed by a solid yellow line displays no Ki67 expression and weak AQP4 expression in arrested glioma cells. **D:** Quantitative analysis of synchronous Ki67 and AQP4 expression revealed the same trend (right, n = 50, ROIs from 13 glioma patients), mean ± SEM, **** *p* < 0.0001. **E:** AQP4 (green) synchronous changes with Ki67 (red) in U87MG cells. The arrowhead indicates that AQP4 and Ki67 were both expressed, and the arrow shows no Ki67 expression and no or weak AQP4 expression. **F:** Further quantitative analysis of Ki67 and AQP4 synchronous expression (60 ROIs from 7 dishes of cell cultures, unpair t-test, ****p < 0.0001). **G:** Ki67 expression in the normal C6 cell lines (Left, Control) and C6 AQP4-KO cell lines (Right, KO), Scale bar, 50 µm. **H:** AQP4-KO glioma Ki67^+^ were significantly lower than those of the control group. Unpaired t-test, n = 6, 16; ****p < 0.0001. Bar height and error bar width represent the mean and SEM, respectively.

To explore whether dynamic AQP4 regulation during the cell cycle is the potential molecule connecting MRI-*k*_io_ with proliferation activity, we also stained AQP4 in human glioma biopsy sample and found that samples with higher Ki67 expression exhibited more pronounced AQP4 expression (**Figure 8A**). Pearson correlation analysis demonstrated a strong positive correlation between the quantitative Ki67^+^ and AQP4^+^ fractions (**Figure 8B**, *r* = 0.91, *p* < 0.0001). In the rat glioma model, we also found the positive linear correlation between the ROI-averaged AQP4^+^ and Ki67^+^ fractions (**Figure S4A, B,*** r* = 0.73, *p* < 0.0001). Furthermore, simultaneous fluorescence staining of AQP4 and Ki67 also revealed that Ki67^+^ cells are more likely to have upregulated AQP4 (**Figure 8C**). The percentage of AQP4^+^ cells was significantly greater than that of AQP4^-^ cells (**Figure 8D,** 70.8% ± 3.6% vs. 29.2% ± 3.6%, *p* < 0.0001, n = 50), whereas the percentage of AQP4^+^ cells was much smaller in the Ki67^-^ population than in the Ki67^+^ population (**Figure 8D,** 11.7% ± 1.8% vs. 70.8% ± 3.6%, *p* < 0.0001, n = 50).

The upregulated AQP4 expression during proliferation was also observed in the U87MG cell cultures. Under confocal microscopy and using multiple fluorescence labeling (**Figure 8E**), the percentage of AQP4^+^ cells were significantly greater than that of AQP4^-^ cells (**Figure 8F**, 85.9% ± 1.8% vs 13.0% ± 2.1%, n = 60, *p* < 0.0001) in the Ki67^+^ cells in U87MG cell cultures. In AQP4^+^ glioma cells, the percentage of Ki67^+^ cells were also significantly greater than that of Ki67^-^ cells (**Figure 8F,** 85.9% ± 1.8% vs 39.4% ± 2.6%, *p* < 0.0001, n = 60).

To validate the involvement of AQP4 as a crucial molecule in regulating glioma proliferation, we created an AQP4-KO C6 cell line using a small guide RNA (sgRNA; Methods). Ki67 expression (Fig. 8G, H) were downregulated with C6 AQP4-KO groups (Ki67^+^ = 16.6% ± 1.0%, n = 16) compared with those of the control with normal C6 cell lines (Ki67^+^ = 53.6% ± 4.2%, n = 6, *p* < 0.0001). This trend was consistent *in vivo*. AQP4-KO significantly reduced Ki67 positivity (Ki67^+^) in rat glioma models (**Figure S4 C, D,** 4.4 ± 1.5% Vs 19.9 ± 3.8% *p* = 0.0007, n = 11, 9). Taken together, AQP4 expression were observed in most proliferating glioma cells and the knockout of AQP4 could largely slow down proliferation activity, suggesting AQP4 is the potential molecule connecting MRI-*k*_io_ with proliferation activity.

## Discussion

*In vivo* mapping of the intratumoral heterogeneity of proliferation activity is crucial for the precise diagnosis and treatment of glioma. In this study, we demonstrated that proliferation activity could largely accelerate the transmembrane water transport, and *k*_io_, a single variable derived from water-exchange DCE-MRI, can precisely detect the dynamic changes of proliferation activity in growth cycle and following TMZ treatment and reveal the intratumoral and intertumoral spatial heterogeneity of proliferation activity in both animal models and human glioma. Upregulated AQP4 expression were observed in most proliferating glioma cells and the knockout of AQP4 could largely slow down proliferation activity, suggesting AQP4 is the potential molecule connecting MRI-*k*_io_ with proliferation activity. More importantly, in clinical practice, water-exchange DCE-MRI can be used together with conventional MRI protocols for glioma diagnosis by adding a water-exchange DCE-MRI sequence during contrast agent injection without additional financial or time costs, making it can be easily adapted for clinical research or application.

Several studies have attempted to predict Ki67 expression using MRI and machine-learning algorithms^15^-^18^. However, most of these studies compared their predictions with the Ki67 level of large surgical samples, indicating an inability to predict proliferation heterogeneity within tumors. A recent study used 52 stereotactic biopsies from glioma patients 2 and found that a combination of four MRI modalities (T2w, fractional anisotropy, cerebral blood flow, and *K*^trans^) with the random forest algorithm could predict the histology-Ki67^+^ with an *R*^2^ value of 0.75. In addition to achieving a much higher *R*^2^ (= 0.89) using the single parameter *k*_io_, our approach has several advantages. First, *k*_io_ has a clear physiological mechanism directly linked to proliferation, whereas other MRI modalities lack such a clear mechanism. Specifically, unlike ADC or DWI, *k*_io_ is insensitive to the cellular microstructure changes since the cell volume or density change has been independently characterized by another pharmacokinetic parameter, *p*_i_. Second, multimodality MRI is susceptible to substantial variability in MR image data across vendor platforms, sequence parameters, and institutions. In contrast, *k*_io_ is a quantitative and physical parameter describing transmembrane water transport. Third, our method only requires the acquisition of DCE-MRI data; multimodality MRI requires three additional modalities (DCE, DTI, and ASL) except for standard MRI, which costs longer acquisition time.

The positive and strong correlation between MRI-*k*_io_ and histology-Ki67^+^ is consistent with the biological premise that rapid cell division relies on increased transmembrane transport of water to maintain cellular homeostasis 9. Using cell sorting, we previously found that human glioma subregions with faster transmembrane water-exchange rates contained more fast-cycling cells and fewer slow-cycling cells relative to subregions with slower transmembrane water-exchange rates 11. In the present study, we confirmed that AQP4 was upregulated in proliferating glioma cells and explains the strong correlation between MRI-*k*_io_ and histology-Ki67^+^. Similarly strong correlations between aquaporin expression and cellular proliferation have also been observed in other tumors (*e.g.*, AQP1 in lung cancer 19 and AQP5 in breast cancer 20).

In this study, we also observed a significant but weak correlation between DWI and Ki67 (*r* < 0.4). This is reasonable because DWI primarily reflects cell density 21, and rapid tumor cell proliferation typically leads to an initial increase in cell density. However, high cell density can also suppress cell proliferation when the tumor microenvironment cannot provide sufficient energy support. Furthermore, DWI is not specific to cell density and can be altered by other cellular factors, such as swelling or shrinking 22. On the other hand, the steady-state cellular water efflux rate *k*_io_, a physical parameter characterizing the membrane permeability property of cells obtained from water-exchange DCE-MRI, is robust in the presence of variations in these microstructural parameters. Three other parameters in water-exchange DCE-MRI (*K*^trans^, *p*_b_, and *k*_bo_) reflecting vascular properties also were significantly but weakly correlated with histology-Ki67^+^. This relationship is expected because vascular proliferation and higher vascular leakage have been identified as biomarkers of malignant proliferating tumors 23,24, but the vessel regulation is heterogenous during tumor development 25. The negative correlation between MRI-*k*_bo_ and Ki67^+^ could potentially result from the redistribution of AQP4 from perivascular astrocytic end-feet to the cell membrane in gliomas 26,27.

Our proposed method could enable the visualization of Ki67 expression *in vivo*. This is particularly important for tumors such as gliomas, which exhibit heterogeneous Ki67 expression. Presurgical Ki67 mapping could help to locate “hot spots” of Ki67 expression, allowing surgeons to obtain optimal biopsies for accurate glioma diagnosis and grading, this would avoid random biopsy selection 28. Additionally, Ki67 expression is potentially useful in predicting the treatment response. However, biopsy acquisition at each follow-up is clearly not feasible. Ki67 mapping could help monitor changes in spatial Ki67 expression *in vivo* during each follow-up tumor assessment. This method has the potential to provide clinicians with evidence-based treatment strategies.

### Limitations

Our study had several limitations. First, the dataset size was small, although it still yielded statistically significant results and correlations. Second, water-exchange DCE-MRI offers the advantage of high spatial resolution but is limited to use in tissues with sufficient contrast agent leakage. A higher dosage of contrast agent administrated could result more accurate estimation of water exchange, whereas a lower dosage could result in a less accurate estimation. Diffusion-based measurements, such as diffusion exchange spectroscopy 29-31, are potential candidates for *k*_io_ measurements with full-brain coverage. Lastly, the integration of *k*_io_ with other imaging modalities, such as PET, could provide a comprehensive assessment of glioma biology, potentially enhancing the broader understanding of tumor heterogeneity and treatment response.

## Conclusion

The strong correlation between *k*_io_ and Ki67^+^ in the dynamic changes and spatial variations of proliferation activity, along with its predictive power in machine learning models, positions *k*_io_ as a promising MRI biomarker for the non-invasive spatiotemporal assessment of glioma proliferation. The mechanism that underlies the potential for *k*_io_ to serve as a direct and accurate biomarker for Ki67 involves the upregulation of AQP4 expression during glioma cell proliferation.

## Materials and Methods

### Water-exchange DCE-MRI measurements of cell cultures with benchtop MRI system

U87MG or C6 cell lines were obtained from the American Type Culture Collection and routinely cultured in Dulbecco's modified Eagle's medium (Sigma-Aldrich, ShangHai D6429-500ML) supplemented with 10% fetal bovine serum (Biological Industries, Israel, 04-002-1A) and 1% penicillin and streptomycin (Gibco, Thermo Fisher Scientific, 10378016) in a humidified incubator. U87MG cells were disassociated with 0.25% trypsin-EDTA solution immediately before MRI scanning, then resuspended in 0.2 mL of phosphate-buffered saline (PBS; or PBS supplemented with 5 mM gadoteridol) in an MRI tube. We then centrifuged the MR tubes at 300 ×*g* at 4°C for 2 min before performing MRI scans. Living cell pellet cultures from the incubator were kept in PBS in a 5% CO_2_ + 95% O_2_ environment to ensure a high cell survival rate. The sample cell pellets from the tubes were mounted into the center of the 0.5 T benchtop MRI system (Pure Devices GmbH) with a fine-thread adjustable rubber ring.

The detailed water-exchange DCE-MRI measurements of the cell cultures are provided by Jia *et al.* 11. Briefly, DWI was initially conducted to localize the cell layer presenting lower apparent diffusivity with a single-slice acquisition, a slice thickness of 5 mm, field of view (FOV) 12.8 × 12.8 mm^2^, matrix size 64 × 64, 16 averages, and two *b* values at 10 s/mm^2^ and 2000 s/mm^2^. For the water-exchange DCE-MRI, an inversion-recovery prepared turbo-spin-echo (IR-TSE) sequence was utilized with an extracellular Gd-based contrast agent. The scanning parameters included echo time (TE) 3ms, turbo factor 16, FOV 12.8 × 12.8 mm^2^, and matrix size 32 × 32. The water-exchange DCE-MRI utilized two CA concentrations (0 and 5 mM) with IR delays and repetition time (TR) adjusted accordingly. The normalized IR-TSE signal was analyzed using a two-site-exchange (2SX) model^13^, accounting for the mole fraction of water in intra- and extracellular compartments and the steady-state intracellular water-efflux rate *k*_io_ (Details are provided in **Supplementary Methods**). After the MRI scans were complete, the samples were immediately fixed with 4% paraformaldehyde for Ki67 immunofluorescence analysis. The growth speed was calculated using the following formula: growth speed (*ct*) = [Num (*ct*) - Num (*ct*-1)]/Dt, where *ct* is time point of cell counting and *ct* - 1 is the previous time point of cell counting, and Num(ct) is the number of cells in the time point, Dt is time gap between the ct and ct - 1 (hours). To obtain the number of cells (Num), we averaged the results from three randomly selected field of view near the center of the culture dish.

### Rat models of glioma and MRI scan

The local Internal Evaluation Committee for Animal Welfare and Rights approved all animal experimental protocols. C6 glioma cells were implanted under the loose skin the right thighs of male Sprague-Dawley rats (8 weeks old). During glioma cell introduction, the rats were anesthetized with a mixture of 2% (v/v) isoflurane in air (R500IE, RWD Life Science Co., Ltd.), and 100 μL PBS containing 5 × 10^6^ C6 cells was subsequently injected into the right leg. Seven to nine days after tumor implantation, the rats underwent MRI scanning on a MAGNETOM 7T system (Siemens Healthcare, Erlangen, Germany) equipped with a surface coil for small-animal imaging. Anesthesia was maintained using a breathing mask within a customized chamber to minimize motion artifacts. Prior to water-exchange DCE-MRI, quantitative T1 and B1 field maps were acquired with specific imaging parameters, T1 mapping was performed using an inversion recovery prepared fast spin-echo (FSE) sequence with: slice thickness 1.5 mm, matrix size 128 × 64, resolution 0.5 × 0.5 mm, and seven IR delays (60, 100, 300, 700, 900, 1200, 1500 ms) with TR = 5000 ms and TE = 8.2 ms was employed. B1 Field Map: Estimated using a multi-flip-angle multi-echo gradient echo (MGE) sequence with TR = 101 ms, TE = 2.8, 6.2, 10.2 ms, and flip angles (FA) = 5°, 7°, 10°, 15°, 20°, 40°, 60°, 70°, 80°, 90°.

The water-exchange DCE-MRI was performed using an MGE sequence with the same spatial settings as the T1 and B1 mapping, with TE = 2.8, 6.2 and 10.2 ms, TR = 101 ms. FA = 20° was used. A total of 220 frames were acquired over 23.7 minutes. A dual-bolus intravenous injection protocol (0.25 mmol/kg at the 20th and 120th frames) was employed to enhance sensitivity to *k*_io_. We utilized the same 3S2X model as the glioma patients to fit the water-exchange DCE-MRI data in rat. This analysis yielded five key parameters: *p*_b_, *p*_o_, *K*^trans^, *k*_bo_, and *k*_io_ (pharmacokinetic interpretation sees** Methods,** Glioma Patients and MRI scan). The detailed water-exchange DCE-MRI analysis of the rats are provided by **Supplementary Methods** and Jia *et al.* 11. The tumors were quickly removed and fixed for IHC after MRI. The maximal tumour size permitted by the institutional review board is 4000 mm^3^ and this limit was not exceeded in this study. All experimental protocols for animal studies were approved by the Animal Experimentation Committee of Zhejiang University (approval number: ZJU20200151).

### Region of interest (ROI) selection for rat glioma model

As most subcutaneous glioma tumours show a ring-shape of high-Ki67 expression, we used concentric donut-shape ROIs to divide both the MRI tumour regions and the corresponding histology images into six ROIs. Then the ROI-averaged *k*_io_ values and Ki67-positive (Ki67^+^) fraction were calculated for each ROI. First, we found the minimum rectangle enclosing the entire tumour and determined the rectangle centre coordinates (X_R_, Y_R_), length L_R_, and width W_R_. Second, a series of concentric oval curves dividing the tumour into six ROIs were automatically drawn.



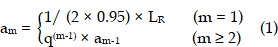





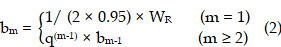



where m was the serial number of each curve (increasing in value from the outer (m = 1)) to the inner (m = 6) and q = 0.75.

### Glioma patients and MRI scan

This was an observational, institutional review board-approved clinical trial, compliant with the Declaration of Helsinki principles (NSFC: NO. 2021-012). **Table S2** provides the information on the adult patients included in this study, according to the STARD guidelines. One or two days before the stereotactic biopsy procedure, MRI data were acquired for each participant using a 3.0 T MRI scanner (Magnetom Skyra, Siemens Healthcare, Erlangen, Germany) equipped with a 20-channel transmit/receive head and neck combined RF coil. The standard MRI protocol included T2-weighted (T2w) three-dimensional (3D) imaging, DWI, and CE T1w MRI. More details of these standard MRI protocol was provided in **Table S4**. The DWI and ADC map was acquired using the RESOVLE sequence with parameters: FOV = (230 mm)^2^, slice thickness = 5 mm, 19 axial slices, in-plane resolution 1.4 × 1.4 mm^2^, δ/Δ = 12 ms/32 ms, TE/TR= 66 ms/4020 ms, b = 0 s/mm^2^ (single acquisition) and 1000 s/mm^2^ (three or six directions, single acquisition in each direction). DWI was calculated by averaging all images of six directions and ADC was calculated using b = 0 s/mm^2^ images and averaged images of b = 1000 s/mm^2^ across all directions by using the following formula (*b*_1_ = 0 s/mm^2^, and *b*_2_ = 1000 s/mm^2^).



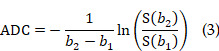



Water-exchange DCE-MRI data were acquired using a gradient echo sequence with a 3D CAIPIRINHA-Dixon-TWIST, volume-interpolated technique with a temporal resolution of 4.5 s and voxel size of 0.9 × 0.9 × 1.5 mm^3^. The flip angle (FA = 10°) was optimized to enhance the sensitivity of the water-exchange DCE-MRI signal to transmembrane water exchange 12. 0.1 mmol/kg Gd-DTPA was administered over 48 s, followed by a 15-mL saline flush with an injection velocity of 2.0 mL/s. Prior to water-exchange DCE-MRI, a double FA MRI sequence 32 was acquired to obtain the pre-contrast T1 map, which used the same sequence and setups as water-exchange DCE-MRI except for the flip angles (FA = 3^o^ and 15^o^). The water-exchange DCE-MRI protocol scan time was approximately 8.7 min. Details are provided in **Table S4 and Table S5.**

The ROI-averaged water-exchange DCE-MRI data was further analyzed using the three-site two-exchange (3S2X) model 12, which was specifically developed for brain tumors to calculate the cellular water efflux rate constant (*k*_io_) and other pharmacokinetic parameters. The 3S2X model considers each tissue voxel to be composed of three compartments: blood (b), extracellular extravascular (o), and intracellular (i) spaces and water exchange happens between blood and extracellular space, and between extracellular and intracellular space. This model has five independent physiological parameters: *p*_b_ and *p*_o_ (water mole fraction for blood and extracellular extravascular space), *k*_bo_ (steady-state water molecule extravasation), *k*_io,_ and *K*^trans^ (contrast agent extravasation rate constant).

### Stereotactic biopsy procedure and paired matching between MRI metrics and biopsy histology

This study was approved by our Institutional Review Board (LCYJ: NO. 2019-103). The stereotactic biopsy was performed by two experienced neurosurgeons within the Leksell Stereotactic System (Elekta, Stockholm, Sweden). A high-resolution CT scan (PHILIPS, Ingenuity Core128) was performed on the patients with the stereotactic head frame fitted. The region of interest (ROI) for each biopsy—a cylinder with a diameter of 2 mm and height of 10 mm was precisely marked on each CT image. Next, CT images were registered to the baseline water-exchange DCE-MRI 3D data via rigid transformation with FLIRT in FSL software 33. The ROI of each biopsy was then converted to water-exchange DCE-MRI space. Within each ROI, the water-exchange DCE-MRI signal intensity was averaged, then subjected to pharmacokinetic modeling and analysis, other MRI metrics was also averaged. This post-processing step allowed for a "point-to-point" comparison between the MRI metrics and the histological Ki67 index obtained from immunohistochemistry (IHC) (**Figure 5A, C**).

### Histopathological and IHC quantification of proliferation

IHC staining of the biopsies was performed using paraffin-embedded sections that had been serially sectioned at 4-μm intervals. Tissue slide images were fully scanned at 20× magnification using a Virtual Microscopy Slide Scanning System (VS120, Olympus, Japan). The scanned images were assessed using ImageJ 34, then analyzed using MATLAB 2019a to remove background signals and quantify both the number of nuclei and the percentage of positive Ki67 or AQP4 staining. Three investigators, blinded to the MRI results, independently quantified the fraction of Ki67-positive cells (Ki67^+^) on IHC images. IHC antibody information is provided in **Table S6**.

### Machine learning models of predicting Ki67 expression in human glioma

Several popular machine-learning algorithms, including the linear and multiple linear regression, regression trees, support vector machines and random forest, were tested. A five-fold cross-validation strategy was used 14, which partitioned the 79-biopsy dataset almost equally into five subsets. The models were trained on a combination of four subsets and validated on the remaining one, a cycle that was repeated five times to ensure each subset was used for validation once. This methodology enabled us to gauge the models' ability to generalize and their sensitivity to how the data was divided. The analyses were conducted using MATLAB 2019a Regression Learning Toolbox.

### Ki67 prediction mapping

To generate quantitative maps estimating Ki67 expression, we utilized the results based on random forests (the most effective approach in our dataset) trained from four different dataset. The prediction variable sets comprised a single variable, *k*_io_; a 4-variable set of standard MRIs; a comprehensive 5-variable water-exchange DCE-MRI set, encompassing all parameters (*p*_b_, *p*_o_, *K*^trans^, *k*_bo_, and *k*_io_); and a 4-variable set of water-exchange DCE-MRI parameters, excluding *k*_io_. We estimated Ki67 expression for each voxel within tumor using a leave-one-patient-out cross-validation scheme 2. This approach most closely simulates a clinical implementation of the algorithm while ensuring the independence of the resulting maps, thereby utilizing all available data.

### Statistics

Statistical analysis was performed with MATLAB 2019a and GraphPad Prism 8.0.2 (GraphPad Software). For comparisons involving multiple groups, ANOVA with Tukey's multiple comparisons test was employed to determine significant differences among groups. For comparisons between two independent groups, unpaired Student's t-tests were used. If the data did not exhibit a gaussian distribution, nonparametric tests were employed instead. Correlation analysis was performed with Pearson's correlation analysis. *p* < 0.05 was considered statistically significant. No animals or data points were excluded from the analyses unless specifically mentioned. Investigators were blinded to group allocation during the experiments and MRI data processing. Careful randomization was conducted when two or more groups were compared. The entropy of the predicted Ki67^+^ imagery, assessed using MATLAB 2019a toolbox (the imhist function), serves as a metric to quantify the heterogeneity present within the Ki67^+^ maps.

## Supplementary Material

Supplementary figures and tables.
