# Supplementary material for: In vivo spatiotemporal mapping of proliferation activity in gliomas via water-exchange dynamic contrast-enhanced MRI
Source: Theranostics. 2025 Mar 24;15(10):4693–707. doi: 10.7150/thno.108479 (PMC11984403; doi:10.7150/thno.108479)
Supplement: Supplementary file 1 — Supplementary figures and tables. [file thnov15p4693s1.zip › 108479w3_Supplementary materials.pdf]

Supplementary Information

Supplementary Figures

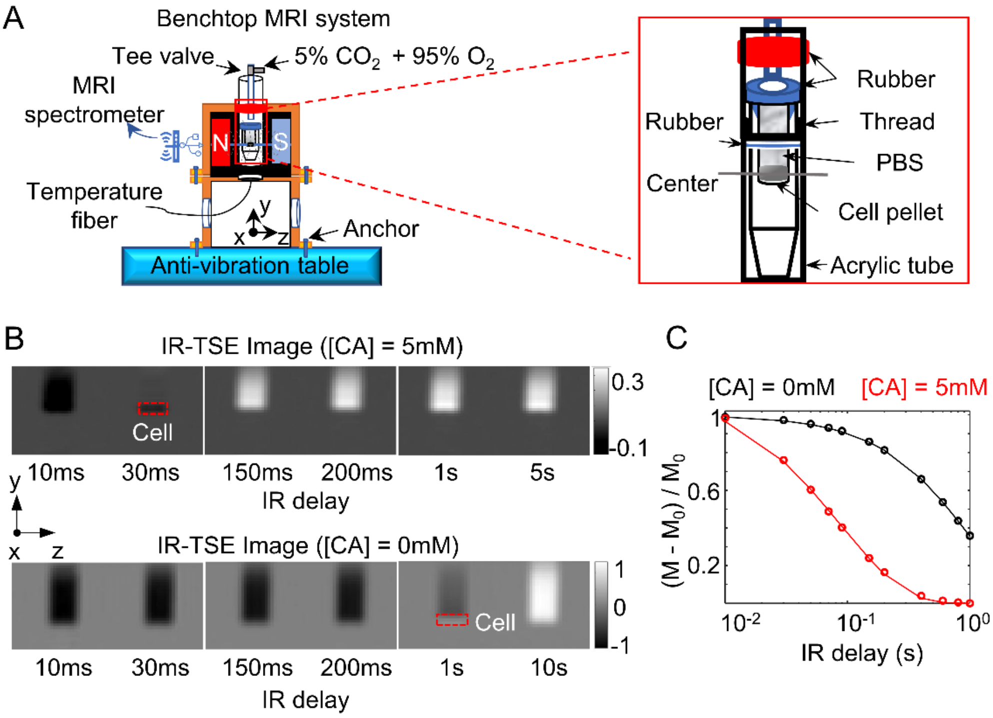

Figure S1. Water-exchange DCE-MRI measurements of cell cultures *in vitro*. A: The benchtop MRI system with a customized cell collection chamber set up for the MRI experiments. **B**: An example of the IR-TSE raw images of U87MG cells at contrast agent (CA) = 5 mM (top) and 0 mM (bottom), the ROI layer of the cell pellet is in the red rectangle. **C**: Example of the normalized IR-TSE signal of U87MG cells (ROI-averaged) at CA concentrations = 5 mM (red**)** and 0 mM (black) in which the circles and continuous curves are the normalized IR-TSE data and the model fitting results with 2SX model, respectively.

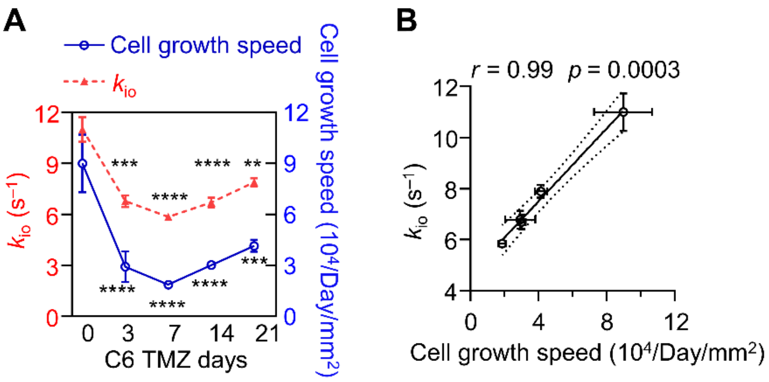

Figure S2. MRI-k_{io} captures the dynamic proliferation activity of C6 cells in response to TMZ treatment. A: The dynamic changes in k_{io} (left Y-axis, red dots, n = 10, 6, 6, 10, 8) and cellular

growth speed (right Y-axis, blue dots, n = 7, 15, 27, 22, 5) as functions of TMZ culture time. Here, the X-axis starts at 0, representing the control group (untreated C6 cells in Log growth phase), Two-way ANOVA Multiple comparisons results between Control (0 day) and TMZ group: *$p < 0.05$, **$p < 0.01$, ***$p < 0.001$, ****$p < 0.0001$. **B:** Correlation analysis between k_{io} and cell growth speed, utilizing data from C6 cells at various TMZ treatment durations (TMZ = 0, 3, 7, 14, 21 days, n = 10, 6, 6, 10, 8), $r = 0.99$, $p = 0.0003$. The solid line indicates the linear regression, while the area between the two dashed curves represents the 95% confidence interval. Here, data are presented as mean ± SEM.

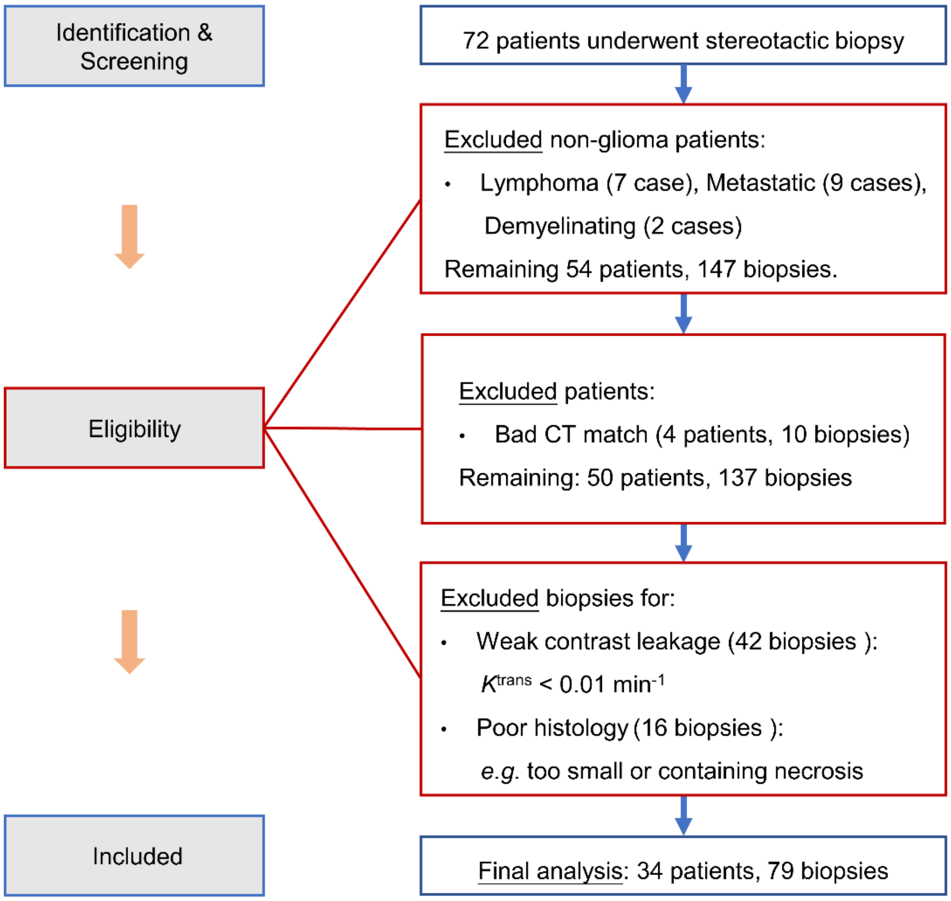

Figure S3. Participant and biopsy selection flowchart.

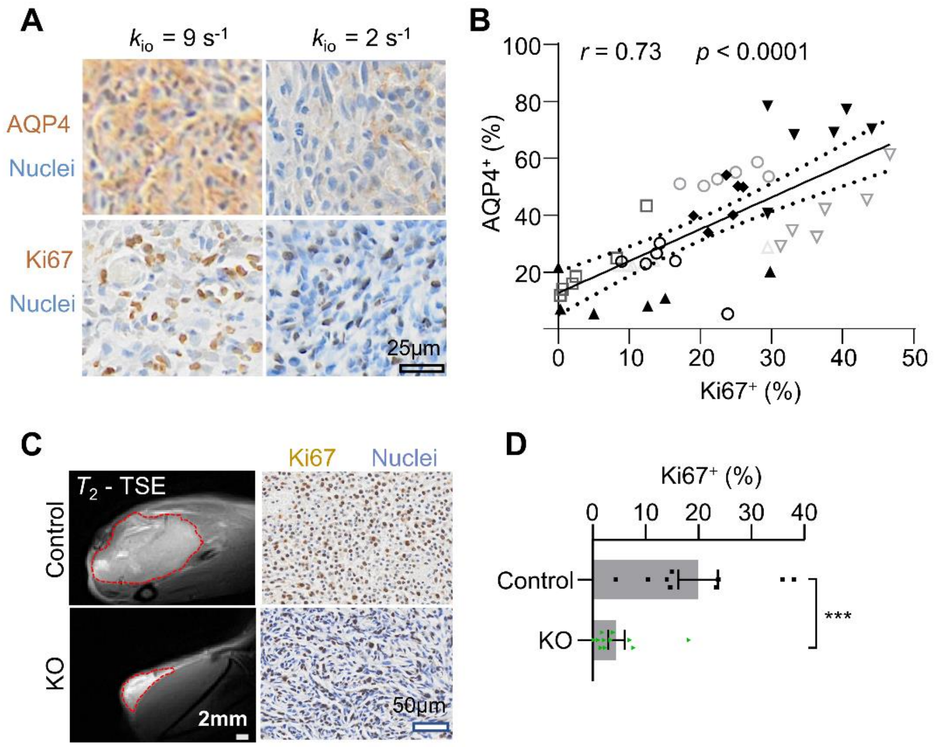

Figure S4. High Ki67 expression ROIs show high AQP4 expression in the rat glioma model. **A**: Typical examples of AQP4 (top) and Ki67 (bottom) expression in rat glioma tissues with high expression (left) and low expression (right) k_{io}; AQP4$^+$ and Ki67$^+$ cells are in brown; nuclei are in blue. **B:** Linear correlation between the ROI-averaged AQP4$^+$ and Ki67$^+$ fractions of 48 ROIs (six ROIs per rat, n = 8), $r = 0.73$, $p < 0.0001$. **C:** The same amount of AQP4-KO C6 cell lines or normal C6 cell lines were subcutaneous injected into rat respectively (see **Methods**). T_2-TSE images of glioma tumor xenografts using normal C6 cell lines (up, Control) and AQP4-KO C6 cell lines (down, KO) on the 9th day after tumor implementation, in which the tumor outlines were illustrated with red dashed lines. Scale bar, 2 mm. **Right**, Typical examples of Ki67 IHC (scale bar, 50 μm) with Control (up) and AQP4-KO (down), respectively. **D**: AQP4-KO glioma Ki67$^+$ were significantly lower than those of the control group. unpaired *t*-test, n = 9, 11; ***$p = 0.0007$. Bar height and error bar width represent the mean and SEM, respectively.

Supplementary Methods

Diffusion MRI and Water-exchange DCE-MRI measurement of cell cultures *in vitro*.

The field strength of the benchtop MRI system used for cell culture measurements was 0.5T. Before Water-exchange DCE-MRI, diffusion weighted imaging (DWI) was performed to localize the cell layer (**Figure 2C**, red rectangle))**:** Single-slice acquisition with a slice thickness of 5 mm, FOV 12.8 × 12.8 mm², matrix size 64 × 64 (zero-filled by a factor of two), 16 averages, and two b-values (10 s/mm² and 2000 s/mm²) were used to localize the cell layer. **Water-Exchange DCE-MRI:** An inversion-recovery prepared turbo-spin-echo (IR-TSE) sequence (**Figure S1B**) was employed with contrast agent (CA), Gadoteridol (Prohance™, Bracco Diagnostics, Inc., Princeton, NJ). Scan parameters included TE = 3 ms, turbo factor = 16, FOV 12.8 × 12.8 mm², matrix size 32 × 32. Measurements were performed at two CA concentrations: 0 mM and 5 mM. At [CA] = 5 mM, 13 IR delays (10 ms, 30 ms, 50 ms, 70 ms, 90 ms, 150 ms, 200 ms, 400 ms, 600 ms, 800 ms, 1 s, 5 s, 5 s) with TR varying together (TR = IR delay + 5 s) were used. For [CA] = 0 mM, the longest IR delays were extended to 10 s with TR extended simultaneously (TR = IR delay + 10 s). Two acquisitions on [CA] = 5 mM were acquired and averaged.

The IR-TSE signal (*M*) at each IR delay was averaged over the cell pellet ROI (**Figure 2C**, red rectangle) and normalized by the equilibrium magnetization (M0, defined as the signal at the longest IR delay). The normalized signal (*e.g.* **Figure S1C**), denoted as $\bar{M}$, was calculated using the formula (1)

$$\bar{M} = (M - M_0)/M_0 \quad (1)$$

A two-site-exchange (2SX) steady-state (SS) model was employed to analyze the normalized IR-TSE signals. This model assumes the MR signal as the sum of contributions from intracellular and extracellular water compartments, each with a distinct R_1 (no cellular leakage). The normalized signal $\bar{M}$ was modeled using a biexponential function,

$$\bar{M} = p_{sm}\exp(-tR_{1sm}) + (1-p_{sm})\exp(-tR_{1lar}) \quad (2)$$

where R_{1sm} and R_{1lar} are the apparently smaller and larger R_1, respectively, and p_{sm} is the apparent fraction of MR signals showing R_{1sm}. The model parameters were determined based on the k_{io}, the R_1 of intracellular water (R_{1i}), and the CA-dependent extracellular water R_1 (R_{1o}). For each

condition (*e.g.*, U87MG at Phase I, II, and III), at least three samples were measured with IR-TSE at [CA] = 0 mM as the baseline data. In this study, R_{1i} were pre-determined by fitting the IR-TSE signals acquired with two different CA concentration on more than three samples in each condition.

Rat glioma models MRI data pre-processing.

For the preprocessing of MRI data from rat glioma models, we employed the trust-region-reflective non-linear least squares fitting algorithm implemented in MATLAB 2019 (MathWorks, Natick, MA, USA). The pre-contrast T1 parametric map was derived by fitting the IR-TSE data using the following equation:

$$M = M_0(1 - (1 - \cos(k * 180°))*\exp(-TI/T_1)) \quad (3)$$

Here, TI denotes the inversion recovery delay, M is the measured MRI signal, and $M0$ and T_1 are fitting parameters. To account for potential B_1 field inhomogeneity, the flip angle (FA) correction factor k was treated as a free parameter. A more precise k map was estimated by fitting the multi-FA MGE data using the equation:

$$S_{(TE=0)} = S_0[1 - e^{-TR*R_1}\cos(k*FA)]^{-1}(1 - e^{-TR*R_1})\sin(k*FA) \quad (4)$$

, where $R_1=1/T_1$, TR is the repetition time, and $S_{(TE=0)}$ (FA) represents the raw T_1-weighted signal estimated from the MGE data at each FA. The MGE signal at a given echo time (TE) was modeled as:

$$S_{(TE)} = S_{(TE=0)} * \exp(-TE/T_2) \quad (5)$$

, $S_{(TE)}$ is the MGE signal at TE, and $S_{(TE=0)}$ and T_2 are the fitting parameters.

3S2X model fitting of both animal and human water-exchange DCE-MRI

For the processing of water-exchange DCE-MRI data, a custom semi-automatic algorithm was used to label voxels showing the fastest increase in signal intensity after CA injection as artery voxels. The signal from these artery voxels was averaged and converted to R1 values using **Eq. (4)**. The CA concentration in plasma (Cp) was then calculated from R1 using the linear relationship:

$$R_1 = R_{10} + r_1(1 - h)C_p \tag{6}$$

where r_1 (mM/s) is the T_1 relaxivity of the contrast agent at 7T, h is the microvascular hematocrit, and R_{10} is the pre-contrast R_1. The CA concentration in the interstitial space at time T, CA_o (T), was characterized using a Kety-Schmidt-type pharmacokinetic model:

$$[CA_o](T) = K^{trans} v_o^{-1} \int_0^T C_p(t) \exp\left(-K^{trans} v_o^{-1} (T - t)\right) dt \tag{7}$$

, where v_o is the volume fraction of the interstitial space and K^{trans} is the CA transfer rate (min^{-1}).

A three-sites two-exchange (3S2X) steady-state (SS) model was employed to fit the T_1-weighted signal of the DCE-MRI data. The model considers the signal as a sum of contributions from three compartments: vascular space (b), interstitial space (o), and intracellular space (i). The system is described by the following matrix equation:

$$\frac{d\mathbf{M}}{dt} = \mathbf{XM} + \mathbf{C} \tag{8}$$

, where the column longitudinal magnetization and relaxation rate vectors are $\mathbf{M} = (M_b, M_o, M_i)$ and $\mathbf{C} = (M_{b0}R_{1b}, M_{o0}R_{1o}, M_{i0}R_{1i})$, respectively, with subscript "0" representing the equilibrium state and R_{1b}, R_{1o}, and R_{1i} represent the longitudinal relaxation rate constants for the blood, interstitial, and intracellular water MR signals in the absence of exchange. Here R_{1o} changes as a function of T and is linearly dependent on $[CA_o](T)$ as **Eq. (6),** and R_{1i} is a constant.

$$\mathbf{X} = \begin{pmatrix} -(R_{1b} + k_{bo}) & k_{ob} & 0 \\ k_{bo} & -(R_{1o} + k_{ob} + k_{oi}) & k_{io} \\ 0 & k_{oi} & -(R_{1i} + k_{io}) \end{pmatrix} \tag{9}$$

Where **X** is the exchange matrix, k_{bo} and k_{ob} denote the steady-state water molecule vascular efflux and influx rate constants, respectively; and k_{io} and k_{oi} denote the steady-state water molecule cellular efflux and influx rate constants, respectively. At equilibrium or steady state (homeostasis), the principle of microscopic reversibility (detailed balance) demands that for the two exchange processes:

$$k_{io}/k_{oi} = p_o/p_i \tag{10}$$

$$k_{bo}/k_{ob} = p_o/p_b \tag{11}$$

To avoid local minimum in non-linear least square (NLLS) fittings, we used a group of initial values for the fitting parameters (**Supplementary Table 6**) and selected the result with the lowest variance

value between fitting data and raw data as the final result. In addition, error analysis was performed on k_{io} maps to remove results with large uncertainties following

$$\chi^2(k_{io}) \geqslant \chi_0^2\left[1 + \frac{K}{N - K}F(K, N - K, 0.95)\right] \tag{12}$$

, where χ^2 was the reduced chi-squared value when performing NLLS with k_{io} fixed at a specific value, χ_0^2 was the reduced chi-squared value with all parameters optimized in NLLS, N was the number of data points in DCE-MRI data, K was the number of free parameters in 3S2X model, and F was the F distribution function. Here the 95% confidence levels were determined and voxels showing low K^{trans} (<0.01min^{-1}) were considered as poor estimation of k_{io} and excluded from the final parametric map.

Supplementary Tables

Table S1. Clinical data for the 34 glioma patients in this study.

Patient ID	Sex	Age (years)	Diagnosis	IHC
patient1	male	57	WHO IV, Recurrent GBM	GFAP+, Olig−2+, S−100+, EMA−, CK−, IDH1−, ATRX+, Ki67+(30%−40%), Vimentin+, p53+(20%), CD34+, NeuN−, CD68−
patient2	female	60	WHO II, Diffuse astrocytic glioma	GFAP+, IDH1−, ATRX+, p53−, Olig−2−, Ki67+(5%)
patient3	male	18	WHO IV, Secondary GBM	IDH1−, ATRX(sporadic+), H3k27M−, S−100+, Olig−2−, GFAP−, NeuN+, Ki67+(30%)
patient4	male	64	WHO I, Pilocytic astrocytoma	Ki67+(3%), Olig−2+, GFAP+, IDH1−, Atrx+, p53−, h3k27m−, CD34+, CD3+, CD20+, CD68+, Neun−
patient5	male	12	WHO III, Recurrent anaplastic ependymoma	GFAP−, Ki67+(20%), S−100−, EMA+
patient6	male	50	WHO IV, GBM	SMA+, Calponin+, CD34−, EMA−, SSTR2−, S−100+, SOX−10−, Olig−2+, GFAP+, Ki67+(10%−30%), Reticulocyte Stain+
patient7	female	55	WHO (III–IV), Recurrent anaplastic ganglioglioma	IDH1+, p53+, GFAP+, Ki67+(40%), Vimentin+, S−100+, CGA+, Neun+, GFAP+
patient8	female	48	WHO IV, Recurrent GBM	GFAP−, ATRX+, IDH1−, Olig−2+, Ki67+(50%), CD34+

patient9	female	32	WHO IV, Secondary GBM	IDH1+, ATRX−, Olig−2+, GFAP+, p53+, Ki67+(40%)
patient10	male	64	WHO III, Anaplastic astrocytoma	GFAP+, Olig−2+, S−100+, IDH1+, p53+, Ki67+(10%), ATRX+
patient11	female	76	WHO II, Anaplastic astrocytoma	CK−, LCA−, GFAP+, S−100+, Ki67+(6%), NeuN−, Olig−2+
patient12	female	52	WHO IV, Secondary GBM	GFAP+, Olig−2+, S−100+, IDH1+, p53+, Atrx+, Ki67+(35%)
patient13	female	67	WHO II, Diffuse astrocytic glioma	CD68+, GFAP+, Olig−2+, NeuN+, ATRX+, p53−, LCA−, CD20−, CD10−, mum−1−, IDH1−, NF+, Ki67+(10%)
patient14	male	19	WHO IV, Recurrent GBM	GFAP+, Olig−2+, S−100+, IDH1−, ATXR−, p53+, Ki67+(60%)
patient15	male	63	WHO IV, GBM	IDH−, KI67+(20%)
patient16	female	45	WHO II, Astrocytic glioma	IDH−, KI67+(10%)
patient17	female	49	WHO IV, Secondary GBM	GFAP+, Olig−2+, IDH1−, ATRX+, p53+(5%), Vimentin+, Ki67+(30%), CD34+, NeuN−, CD68+
patient18	female	33	WHO III, Recurrent GBM	GFAP+, OLIG−1+, ATRX−, IDH1+, p53+
patient19	male	47	WHO IV, GBM	GFAP+, Olig−2+, Ki67+(30%), IDH1−, ATRX+, p53+, CD34+
patient20	female	66	WHO (III–IV), glioma	FAP+(part), Olig−2+(part), p53−, IDH1−, ATRX+, H3K27M−, Ki67+(15%), S−100+, EMA−, Syn+, LCA−, CD3−, CD20−, CK(AE1/AE3)−, NF−

patient21	male	63	WHO IV, GBM	GFAP+, Olig−2−, S−100−, IDH1−, ATRX+, LCA−, p53+, Ki67+(60%), Vimentin+, CK(AE1/AE3)
patient22	female	67	WHO III, glioma	GFAP+, Olig−2+, CD3−, CD20−auto−, PAX5−, BCL6−, MUM−1−, CD10−, Ki67+(20%), p53+(mutant), Syn+, IDH1−, ATRX+, H3K27M−, NeuN−, CD34−, S100−auto+, EMA−, INI−1−, Fli−1−
patient23	female	54	WHO IV GBM	GFAP+, Olig−2+, Vimentin+, IDH1−, ATRX+, p53−, EMA−, PR−, D2−40+, Ki67(70%), SSTR2−
patient24	male	52	WHO III, Anaplastic astrocytic carcinoma	GFAP+, S100−auto+, Olig−2+, ATRX−, IDH1+, p53+(mutant), Ki67+(20%)
patient25	male	62	WHO IV, GBM	GFAP(+weak), S100−auto−, Olig−2+, CD3−, CD20−auto−, CD21−, Ki67+(40%), LCA−, ATRX+, IDH1−, p53−, Syn+, NeuN−, CD34−
patient26	female	74	WHO (III–IV), glioma	GFAP+, Olig−2+, S100−auto+, p53+(mutant), Ki67+(60%), LCA−, IDH1−, ATRX+, NeuN−, CD34−
patient27	female	57	WHO (III–IV), glioma	GFAP(+weak), Olig−2+, EMA−, IDH1−, ATRX+, p53+(part), Ki67+(20%), CK(AE1/AE3)−, Syn−, Vimentin+, INI−1+, NeuN−f P16−, SSTR2−, STAT6−, CD34−, CD68−, CR−, PHH3+(part), LCA−

patient28	male	48	WHO (I–II), glioma	GFAP+, Olig−2+, IDH−, p53−, Ki67+(1%), CD34+, CD20−, LFB+
patient29	female	42	WHO II, glioma	NeuN+, GFAP+, Olig−2+, ATRX+, IDH1−, p53−, Ki67(2%), CD34+
patient30	male	39	WHO IV, diffuse midline glioma, DMG	GFAP+, Olig−2+, CK(AE1/AE3)−, Vimentin+, LCA−, Ki67+(20%), ATRX+, IDH1−, p53+(mut), NeuN−, GFAP +, H3K27M+, S100+, Calretinin−, MAP2abc+, CD34+
patient31	female	62	WHO IV, Recurrent GBM	GFAP+, Olig−2+, SSTR2+, EMA−, PR+, Ki67+(20%), IDH1−, p53+(20%), ATRX+, SOX10−
patient32	male	58	GBM, WHO IV	GFAP+, Olig−2+, IDH1 +(NS), ATRX+, p53+, Ki67+(30%), CR−, CD34+
patient33	female	59	GBM, WHO IV	GFAP+, ATRX−, Ki67+(90%), IDH1(−), CD34(−), NeuN(−), Olig−2(+), EMA(−), LCA(−), CK(AE1/AE3)(−), Syn(+)
patient34	female	59	GBM, WHO IV	GFAP+, Olig−2(+), Ki67+(40%), p53+(mut), IDH1(−), ATRX+

Table S2: Participant Demographics

Characteristic	WHO Grade II	WHO Grade III	WHO Grade IV
No. of patients	7	8	19
No. of men	2 (5)	3 (5)	7 (12)
Age (y)	57 ± 13	52 ± 20	50 ± 14
Astrocytoma	3	2	0
Diffuse astrocytic	4	0	0
Anaplastic ganglioglioma	0	2	0
Oligodendrogliomas	0	3	0
Anaplastic astrocytic carcinoma	0	1	0
Diffuse midline glioma	0	0	1
Glioblastoma	0	0	18

Table S3. Results of several popular machine-learning algorithms and multimodal MRI in predicting Ki67 expression in human gliomas

		Regression Learning			
Model Name		k_{io}	Convention	k_{bo}+P_b+P_o+K^{trans}	water-exchange DCE-MRI
Random forest	R^2	**0.89**	**0.62**	**0.53**	**0.90**
	RMSE (%)	6.9	15.1	15.9	6.7
Liner Regression Models (Linear)	R^2	0.83	0.09	0.05	0.83
	RMSE (%)	8.4	19.9	19.7	8.3
Regression Trees (Fine Tree)	R^2	0.80	0.11	0.39	0.77
	RMSE (%)	9.1	19.7	23.8	9.6
Support Vector Machines (Linear SVM)	R^2	0.80	0.02	0.02	0.83
	RMSE (%)	9.0	21.0	20.0	8.3
Gaussian Process Regression Models (Rational Quadratic)	R^2	0.82	0.08	0.04	0.66
	RMSE (%)	8.5	20.0	20.6	11.8
Ensembles of Trees (Bagged Trees)	R^2	0.81	0.19	0.03	0.80
	RMSE (%)	8.6	18.8	20.5	8.9

R^2, the coefficient of determination; RMSE, root mean square error. The best prediction results are in bold font.

Table S4: Detailed list of MRI sequence parameters in human. (TWIST = Time-resolved angiography With Interleaved Stochastic Trajectories, PGSE = Pulsed Gradient Spin-Echo, CE T1w = post-contrast T1 weight, MPRAGE = Magnetization-Prepared Rapid Gradient-Echo, T2w = T2 weight, TR = repetition time, TE =Echo time, FA = Flip Angle, FOV = Field of View, BW = Bandwidth.

	water-exchange DCE-MRI	T1 mapping	DWI	CE T1w	T2w
Slice orientation	Axial	Axial	Axial	Sagittal	Sagittal
Pulse seq. Name	TWIST	TWIST	PGSE	PRAGE	SPACE
TR (ms)	6	6	4020	2300	3200
TE (ms)	2.46	2.46	66	2.29	409
b (s/mm^2)	N/A	N/A	0, 1000 (3 or 6 directions)	N/A	N/A
FA (°)	10	3/15	90	8	120
FOV (cm)	34 × 34	34 × 34	23 × 23	24 × 24	23 × 23
Matrix	384 × 384	384 × 384	160 × 160	256 × 256	256 × 256
Slice	80	80	19	192	192
Slice thickness(mm)	1.5	1.5	5	1	1
BW (kHz)	450	450	920	200	725

Table S5. Detailed list of water-exchange DCE-MRI sequence parameters for cultured cells, rats, and humans

IR-TSE = Inversion-Recovery Turbo Spin Echo. MGE = Multi-Flip-Angle Multi-Echo Gradient Echo.

Parameters	Cells (0.5T)	Rats (7T)	Humans (3T)
Pulse seq. Name	IR-TSE	Multi-Flip-Angle MGE	CAIPIRINHA-Dixon-TWIST
Contrast Agent	0mM and 5 mM	0.25 mmol/kg	0.1 mmol/kg
Injection Protocol	N/A	Dual-bolus	Single-bolus
TR	5000 ms	101 ms	6 ms
TE	3ms	2.8, 6.2, 10.2 ms	2.46 ms
FA	180°, 90°	20°	10°
FOV	12.8 × 12.8 mm^2	64 × 32 mm^2	340 × 340 mm^2
Matrix	32 × 32	128 × 64	384 × 384
Slices	1	7	80
Slice Thickness	5mm	1.5 mm	1.5 mm

Table S6. Immunofluorescence (IF) and IHC antibody information

Target	Name	Function	Ratio	Sources
AQP4 (IHC&IF)	Anti-aquaporin 4 antibody	Primary	1:100 (IF); 1:1000 (IHC)	Abcam, ab46182
	Anti-rabbit IgG	Secondary	1:500	cell signaling #4413S
Antibody (IHC)	Horseradish-peroxidase (HRP)	Secondary	1:1000	Servicebio, GB23303
Antibody (IHC)	Anti-mouse IgG	Secondary	1:500	cell signaling #4408S
	Anti-rabbit IgG	Secondary	1:500	Beyotime, A0516
Ki67	Ki67 mouse antibody	Primary	1:200	Servicebio, GB121141 for co-expression
	Anti-Mouse IgG	Secondary	1:500	Beyotime, A0521
